# Cell-specific and athero-protective roles for RIPK3 in a murine model of atherosclerosis

**DOI:** 10.1242/dmm.041962

**Published:** 2020-01-24

**Authors:** Sarah Colijn, Vijay Muthukumar, Jun Xie, Siqi Gao, Courtney T. Griffin

**Affiliations:** 1 Cardiovascular Biology Research Program, Oklahoma Medical Research Foundation, Oklahoma City, OK 73104, USA; 2 Department of Cell Biology, University of Oklahoma Health Sciences Center, Oklahoma City, OK 73190, USA

**Keywords:** Necroptosis, Macrophages, Endothelial cells, MCP-1, Mouse

## Abstract

Receptor-interacting protein kinase 3 (RIPK3) was recently implicated in promoting atherosclerosis progression through a proposed role in macrophage necroptosis. However, RIPK3 has been connected to numerous other cellular pathways, which raises questions about its actual role in atherosclerosis. Furthermore, RIPK3 is expressed in a multitude of cell types, suggesting that it may be physiologically relevant to more than just macrophages in atherosclerosis. In this study, *Ripk3* was deleted in macrophages, endothelial cells, vascular smooth muscle cells or globally on the *Apoe^−/−^* background using Cre-lox technology. To induce atherosclerosis progression, male and female mice were fed a Western diet for three months before tissue collection and analysis. Surprisingly, necroptosis markers were nearly undetectable in atherosclerotic aortas. Furthermore, *en face* lesion area was increased in macrophage- and endothelial-specific deletions of *Ripk3* in the descending and abdominal regions of the aorta*.* Analysis of bone-marrow-derived macrophages and cultured endothelial cells revealed that *Ripk3* deletion promotes expression of monocyte chemoattractant protein 1 (MCP-1) and E-selectin in these cell types, respectively. Western blot analysis showed upregulation of MCP-1 in aortas with *Ripk3*-deficient macrophages. Altogether, these data suggest that RIPK3 in macrophages and endothelial cells protects against atherosclerosis through a mechanism that likely does not involve necroptosis. This protection may be due to RIPK3-mediated suppression of pro-inflammatory MCP-1 expression in macrophages and E-selectin expression in endothelial cells. These findings suggest a novel and unexpected cell-type specific and athero-protective function for RIPK3.

This article has an associated First Person interview with the first author of the paper.

## Abstract (precis)

**Summary:** This study reports that RIPK3 in macrophages and endothelial cells protects against atherosclerosis progression in mice, providing novel information about unexpected roles for RIPK3 in an inflammatory vascular disease.

## INTRODUCTION

Atherosclerosis is a highly complex pathology that is influenced by a multitude of environmental and genetic factors. The cellular mechanisms that have been linked to atherosclerosis development are vast in number and include metabolic imbalances, inflammation, fibrosis, mechanotransduction and cell death (Lusis, 2000; Tabas et al., 2015). Occasionally, these mechanisms are controversial when there are conflicting reports about the role of a particular gene in atherosclerosis. However, in order to safely translate new knowledge into a clinical setting, the benefits and disadvantages of targeting specific genes must be explored.

Cell death is one of the leading causes of inflammation and necrotic core formation in atherosclerosis (Van Vré et al., 2012). Although apoptosis and secondary necrosis occur in a variety of cells in atherosclerotic plaques (Van Vré et al., 2012), recent reports propose that plaque macrophages also undergo necroptosis, a newly identified form of programmed cell death (Lin et al., 2013; Meng et al., 2015; Karunakaran et al., 2016; Leeper, 2016). Necroptosis relies on the essential executioner receptor-interacting protein kinase 3 (RIPK3) and phosphorylation of its downstream effector mixed lineage kinase domain-like protein (MLKL), which then acts to permeabilize the plasma membrane (Dhuriya and Sharma, 2018; Linkermann and Green, 2014). Similar to secondary necrosis, necroptosis culminates with a release of intracellular components that initiate an immune response. This type of programmed death is often associated with immune cells, and several studies show that wild-type macrophages are susceptible to necroptosis, whereas *Ripk3^−/−^* macrophages are protected (Karunakaran et al., 2016; Lin et al., 2013; Meng et al., 2015). As RIPK3 is necessary for necroptosis – and as necroptosis is considered to be inherently inflammatory – researchers have suggested that RIPK3 or MLKL should be targeted to decrease atherosclerosis severity in the clinical setting (Zhe-Wei et al., 2018; Coornaert et al., 2018).

However, more recent work has revealed that RIPK3 has pleiotropic roles beyond necroptosis (Silke et al., 2015; Moriwaki and Chan, 2016; Vince and Silke, 2016; He and Wang, 2018; Weinlich et al., 2016). These new mechanisms include NF-κB-induced cytokine production and NLRP3 inflammasome-induced or caspase 8-induced IL-1β activation, which expand the pro-inflammatory capacity of RIPK3 activity beyond necroptosis. Surprisingly, RIPK3 has also been reported to promote aerobic metabolism through phosphorylation of several metabolic enzymes (Yang et al., 2018), thus it is also possible for RIPK3 to act in a non-inflammatory manner. Overall, these alternative functions for RIPK3 are often unacknowledged in disease studies.

When *Ripk3* is genetically deleted in a murine model of atherosclerosis, one report shows that atherosclerotic lesion area, necrotic area and macrophage infiltration are decreased (Lin et al., 2013). Another report shows that the necroptosis chemical inhibitor necrostatin-1 improves atherosclerosis severity (Karunakaran et al., 2016). However, these reports do not fully explore the pleiotropic roles of RIPK3, and instead propose that RIPK3 causes plaque macrophages to undergo inflammatory necroptosis. Moreover, as necrostatin-1 has many off-target effects and can inhibit apoptosis and necroptosis-independent inflammatory pathways (Vandenabeele et al., 2012), it is a non-ideal inhibitor for examining the specific effects of necroptosis. Furthermore, these studies do not address the fact that different cell types tend to use pro-inflammatory components very differently (Mussbacher et al., 2019), and thus RIPK3 could be playing alternative roles in each of the various cell types of the plaque.

As RIPK3 is a widely expressed protein – as reported by the Human Protein Atlas (Uhlén et al., 2015) – there is potential for RIPK3 to have tissue- or cell-specific functions. To explore the cell-specific function of RIPK3 in the vasculature, and to confirm which cell types – if any – undergo necroptosis in atherosclerosis, we developed a conditional model of *Ripk3* deletion that utilizes a *Ripk3* locus integrated with loxP sites (Colijn et al., 2019). We conducted this study by using the *Ripk3-*floxed mice crossed with macrophage-, vascular smooth muscle cell- and endothelial cell-specific Cre recombinases on the *Apoe^−/−^* murine model of atherosclerosis. This conditional deletion of RIPK3 aids in understanding how cell-specific RIPK3 inhibition affects atherosclerosis and gives insight into the consequences of targeting components of the necroptosis pathway in a disease context.

We now report that RIPK3 plays a biologically relevant role in atherosclerosis in macrophages and endothelial cells through an athero-protective – and likely non-necroptotic – mechanism. Our data indicate that RIPK3 plays an anti-inflammatory role in these cell types, possibly through the suppression of monocyte chemoattractant protein-1 (MCP-1; also known as CCL2) in macrophages and E-selectin (SELE) in endothelial cells. These results provide novel information about unexpected roles for RIPK3 in an inflammatory vascular disease, and raise questions about our previous understanding of the relationship between RIPK3, necroptosis, inflammation and atherosclerosis.

## RESULTS

### *Ripk3* transcripts are present in atherosclerotic plaques at very low copy numbers

**Fig. 1.**
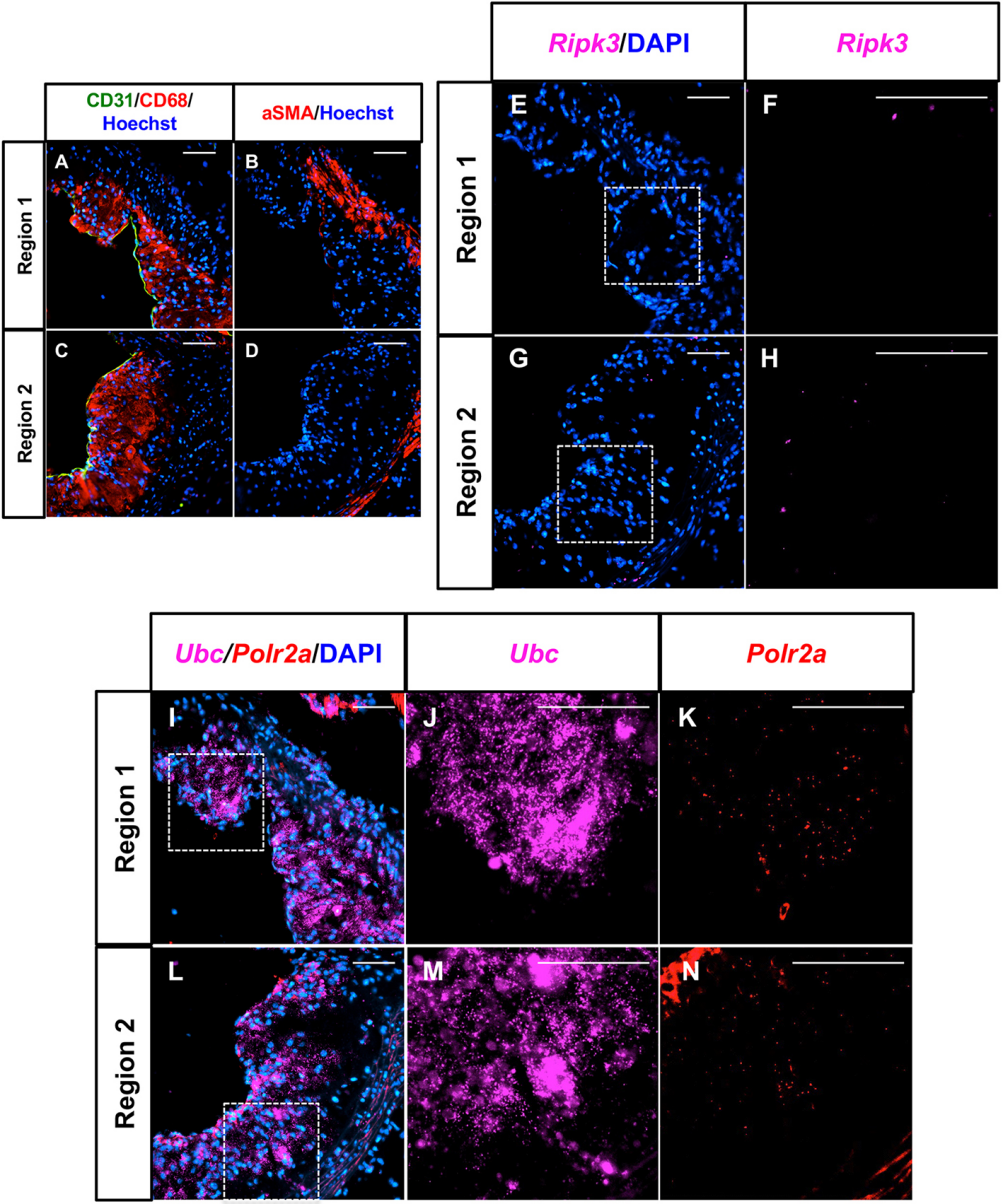
***Ripk3* transcripts are present in atherosclerotic plaques at very low copy numbers.** (A-D) Sequential cross sections of control aortic roots were immunostained for the endothelial cell marker CD31 (green) and macrophage marker CD68 (red) (A,C) or for the smooth muscle cell marker aSMA (red) (B,D) and were co-stained to identify nuclei (Hoechst; blue). Two plaque regions that contain all three cell types are shown. (E-N) RNAScope^®^ RNA *in situ* hybridization was performed on sequential sections of the two separate regions of control aortic roots shown in A-D. *Ripk3* probe was used to identify *Ripk3* transcripts (pink) and co-stained with DAPI (blue) to identify nuclei (E,G). F and H show magnifications of the boxed regions (without DAPI) in E and G, respectively. Positive control probes *Ubc* (pink; high transcript copy number) and *Polr2a* (red; low transcript copy number) were used to show the efficacy of the assay (I,L). J,K and M,N show magnifications of the boxed regions (without DAPI) in I and L, respectively. Positive signal is determined by the presence of ‘dots’ which are each meant to represent a single transcript. Images are representative of *in situ* hybridization experiments performed on sections from three separate control mice (*n*=3). Scale bars: 25 µm.

To explore the role of RIPK3 in the various cell types of atherosclerosis, we first attempted to look at RIPK3 expression in the plaque regions. Unfortunately, as is fairly common for plaque immunostaining, all commercial antibodies that we used to detect RIPK3 showed widespread non-specific staining, which was confirmed by using *Ripk3*-knockout plaques as controls (data not shown). As an alternative to immunostaining, we used RNA *in situ* hybridization with RNAScope^®^ technology to identify the expression pattern of *Ripk3.* After confirmation of the specificity of the *Ripk3* probe (Fig. S1) and after identifying plaque areas with endothelial cells, macrophages and smooth muscle cells, we showed that *Ripk3* transcripts were nearly undetectable in these regions (Fig. 1A-H). In fact, *Ripk3* levels were even lower than *Polr2a* levels, which is a ubiquitously expressed positive control gene that is known to produce very low transcript copy numbers per cell (Fig. 1I-N) (Bingham et al., 2017). This very low copy number for *Ripk3* transcripts prevented dual immunostaining to identify cell types with *Ripk3* expression, as the additional steps would wash away the probe signal; however, we were able to immunostain sequential sections to confirm that all the pertinent cell types were present (Fig. 1A-D). Regardless, it is apparent that *Ripk3* transcript copy number is very low in all cell types present in our atherosclerotic plaques, and that immunostaining is not ideal for detecting RIPK3 protein in plaque tissues. However, as previous studies have already linked RIPK3 and atherosclerosis (Lin et al., 2013; Meng et al., 2015), we suspected that this low *Ripk3* transcript copy number did not accurately predict RIPK3 activity in the aorta.

### RIPK3 deletion in macrophages or endothelial cells exacerbates atherosclerosis

Next, we wanted to determine the biological relevance of cell-specific RIPK3 in atherosclerosis, so we conditionally deleted *Ripk3* in macrophages (*Ripk3Δ^MΦ-Cre^*), smooth muscle cells (*Ripk3Δ^SMC-Cre^*), endothelial cells (*Ripk3Δ^EC-Cre^*) and globally (*Ripk3Δ^Global^*) on the *Apoe^−/−^* background using Cre-lox technology and *LysM* (*Lyz2*)*-Cre*, *SM22* (*Tagln*)*-Cre*, *VE-Cadherin* (*Cdh5*)*-Cre* and germline excision, respectively. For brevity and clarity**,** simplified nomenclature is used throughout the article in place of the complete genotypes. The full genotypes, associated nomenclature, and important notes are described in Table 1. These mice were then fed a Western diet for three months, at which point the tissues were collected for analysis. It should be noted as a caveat to our study that we did not obtain as many of the *Ripk3Δ^Global^* mice as we did the cell-specific knockouts owing to the difficulties associated with breeding and maintaining the *Apoe^−/−^* lineage.

**Table 1.**
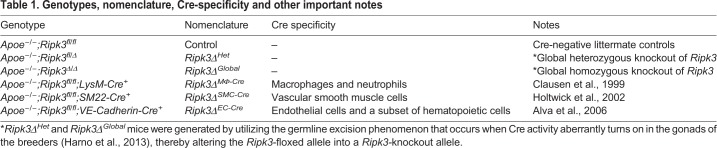
**Genotypes, nomenclature, Cre-specificity and other important notes**

**Fig. 2.**
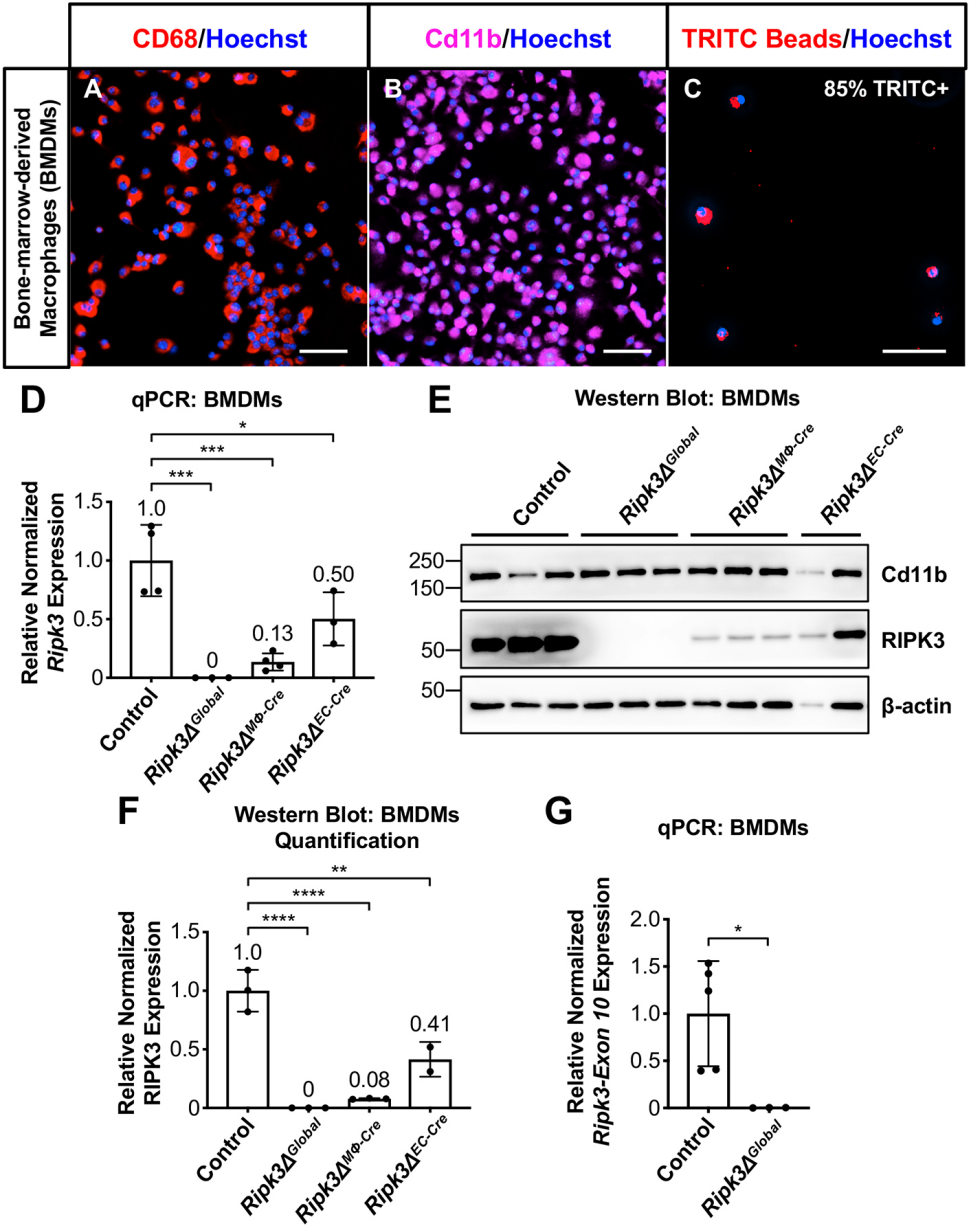
**Bone-marrow-derived macrophages exhibit significant reduction in RIPK3 levels in *Ripk3Δ^Global^*, *Ripk3Δ^MΦ-Cre^* and *Ripk3Δ^EC-Cre^* mice.** (A,B) BMDMs were isolated, differentiated on chamber slides for 7 days, immunostained for macrophage markers CD68 (red; A) and CD11b (pink; B), and co-stained for nuclei (Hoechst; blue). All cells from seven separate BMDM isolations were positive for these markers (*n*=7). (C) A phagocytosis assay was performed on BMDMs with fluorescent polystyrene beads. Phagocytic cells display TRITC^+^/Hoechst^+^ signal, and 85% of cells from two separate BMDM isolations were double positive (*n*=2). (D-F) Protein or RNA was collected from control, *Ripk3Δ^Global^*, *Ripk3Δ^MΦ-Cre^* and *Ripk3Δ^EC-Cre^* BMDMs. RNA was converted to cDNA and analyzed by qPCR for *Ripk3* levels (D). Protein lysates were immunoblotted to identify CD11b, RIPK3 and β-actin (loading control) (E) and quantified (F). (G) RNA from control and *Ripk3Δ^Global^* BMDMs was converted to cDNA and analyzed by qPCR for *Ripk3-Exon 10* levels. For panels D,F,G, each dot represents a BMDM isolation from an individual animal. Statistics for panels D and F were calculated using one-way ANOVA with Dunnett's multiple comparisons test. Overall ANOVA *P*-values (prior to the *post hoc* tests) are 0.0002 (D) and <0.0001 (F). Statistics for panel G were calculated using an unpaired *t*-test with Welch's correction. *P<0.05, **P<0.01, ***P<0.001, ****P<0.0001. Data are mean±s.d. Scale bars: 25 µm.

As *VE-Cadherin-Cre* has been reported to delete in a portion of the adult bone marrow (Alva et al., 2006), and *LysM-*Cre is occasionally inefficient (Clausen et al., 1999), we investigated the efficiency of deletion by isolating bone-marrow-derived macrophages (BMDMs) and analyzing their RIPK3 expression levels (Fig. 2A-F). *LysM-Cre* showed robust deletion efficiency by transcript and protein analysis. As expected, *VE-Cadherin-Cre* showed a partial deletion of RIPK3 in BMDMs as well, meaning that we could not ignore a potential role for *Ripk3-*deficient macrophages in *Ripk3Δ^EC-Cre^* mice.

We also sought to confirm that the *Ripk3*-floxed allele – which results in removal of the first nine out of the ten exons of *Ripk3* after Cre-mediated excision (Colijn et al., 2019) – does not produce a transcribed version of exon 10. This exon encodes a functional domain that is involved in several kinase-independent processes (Moriwaki et al., 2017), which, if still translated, could confound our results. However, transcript analysis using primers designed specifically for exon 10 show that there is no possibility for translation of a truncated form of RIPK3 (Fig. 2G). Furthermore, *Ripk3Δ^Global^* aortas show no detectable RIPK3 protein by immunoblotting (Fig. S2), altogether indicating that we were able to generate a true knockout allele.

**Fig. 3.**
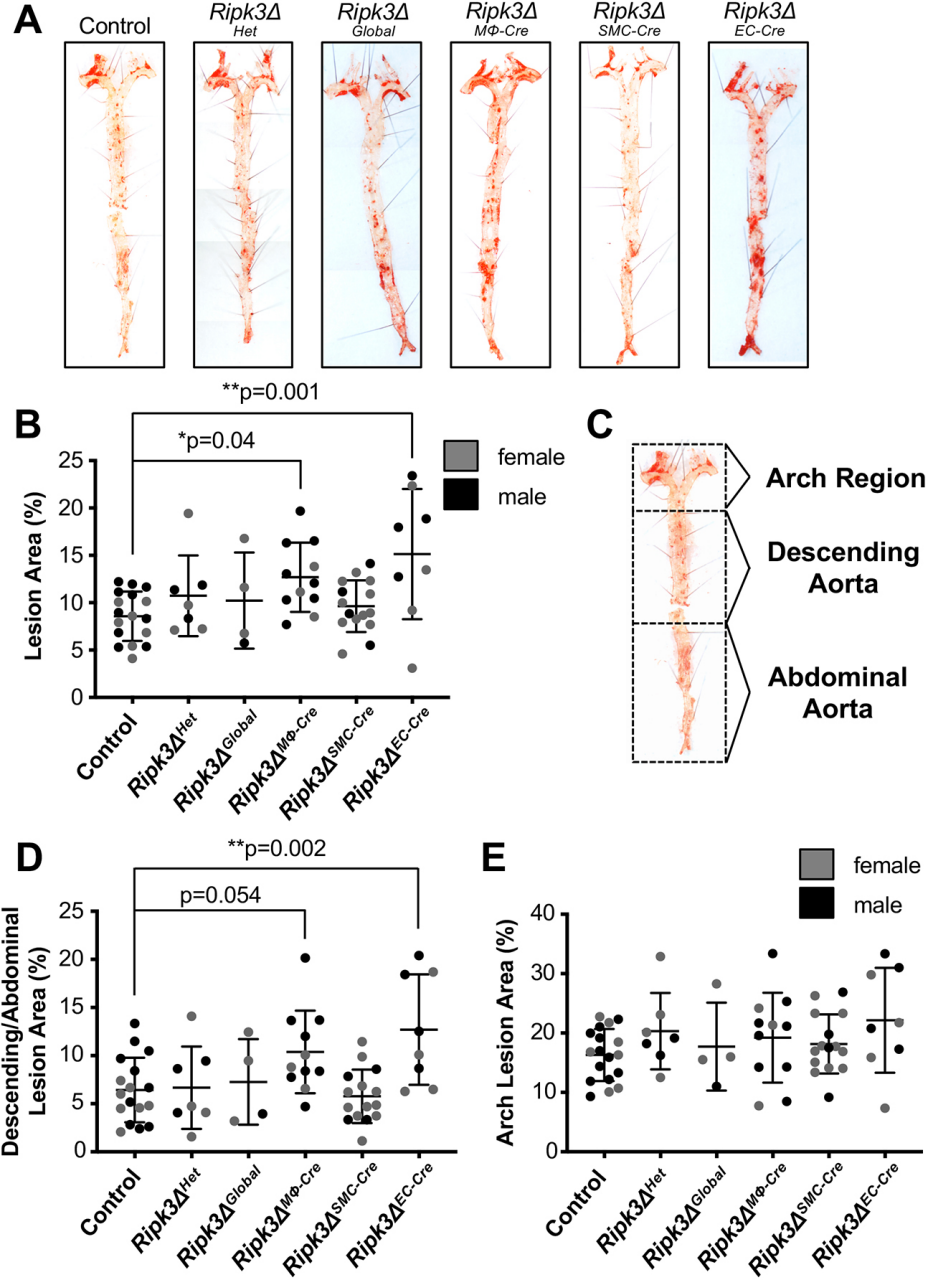
***Ripk3Δ^MΦ-Cre^* and *Ripk3Δ^EC-Cre^ en face* aortas exhibit increased lesion areas in the descending and abdominal aortic regions.** (A) Representative images of control and *Ripk3*-deficient aortas from male and female mice that were fed the Western diet for 3 months. Aortas were then dissected, cut open *en face* and stained with ORO to visualize lesions. (B) Lesion area was quantified as the percent lesion area of the whole aortic area. (C) Aortic areas were analyzed separately and divided into the arch, descending and abdominal regions. The arch region extends from where the aorta enters the heart to the end of the curvature. The descending region extends to the renal arteries. The abdominal region extends to the iliac arteries. (D,E) Lesion area was quantified for the descending and abdominal regions (D) and arch region (E). For panels B,D,E, each dot represents an aorta from an individual animal. Statistics were calculated using one-way ANOVA and Dunnett's multiple comparisons test for panels B and D. Overall ANOVA *P*-values (prior to the *post hoc* tests) are 0.005 (B), 0.001 (D) and 0.33 (E). Data are mean±s.d.

If previous reports are correct that RIPK3 increases atherosclerosis severity and that necroptotic macrophages are the main source of that increase in severity, then we would expect to see less atherosclerotic phenotypic severity in the *Ripk3Δ^Global^*, *Ripk3Δ^Het^*, and *Ripk3Δ^MΦ-Cre^* mice. *En face* Oil Red O staining revealed that *Ripk3Δ^MΦ-Cre^* and *Ripk3Δ^EC-Cre^* aortas showed an increase in percent atherosclerotic lesion area, whereas *Ripk3Δ^Het^* and *Ripk3Δ^Global^* aortas were no different from control, which conflicts with the previously published genetic study addressing the role of RIPK3 in atherosclerosis progression (Fig. 3A,B) (Lin et al., 2013). Instead, our data suggest that RIPK3 plays an athero-protective role in macrophages and endothelial cells.

**Fig. 4.**
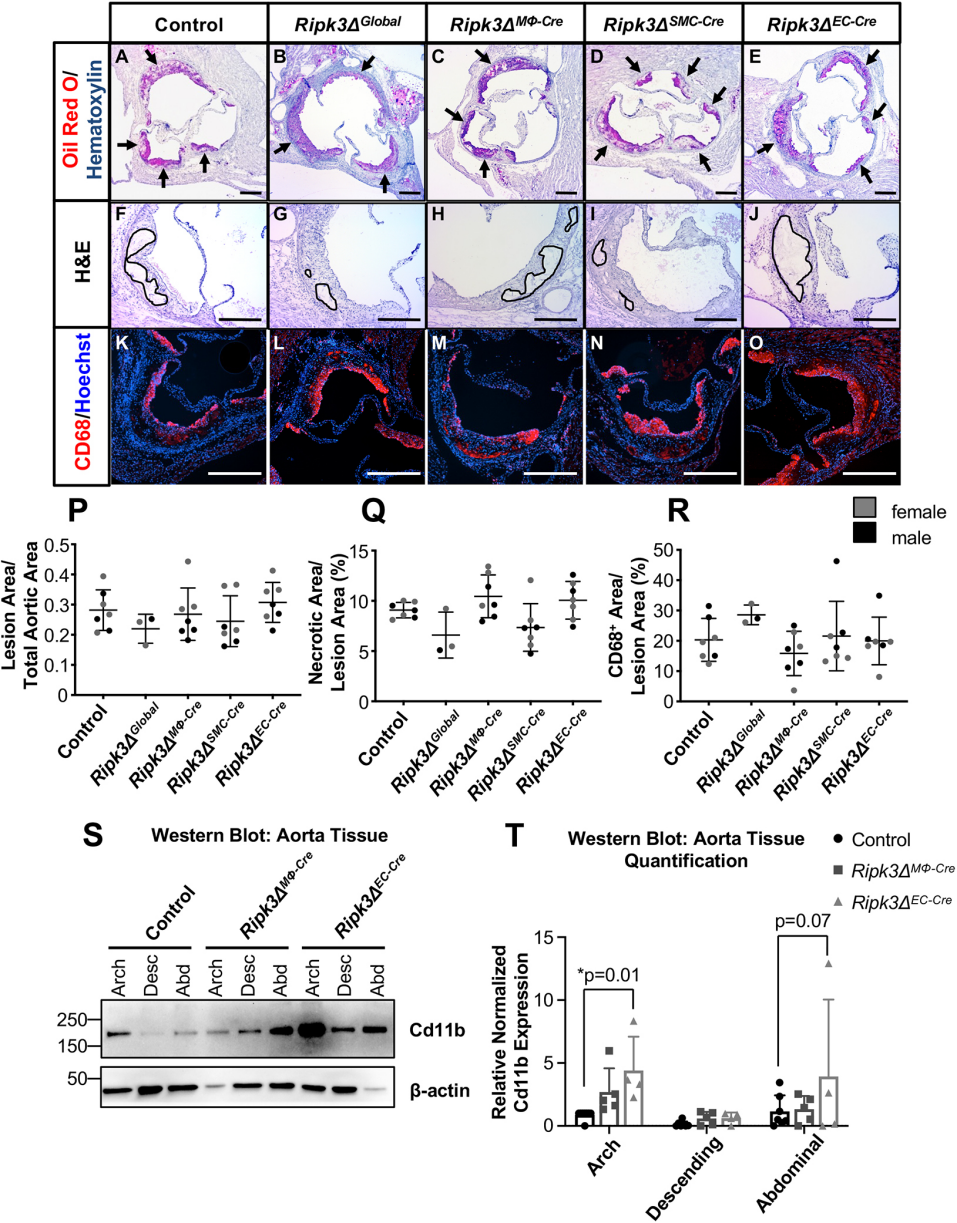
**Aortic root analyses reveal no differences in lesion area, necrotic area or macrophage area, though *Ripk3Δ^EC-Cre^* aortas exhibit higher CD11b signal.** (A-R) After 3 months on a Western diet, hearts were dissected and aortic roots were sectioned and analyzed for lesion area, necrotic area and macrophage area. Aortic roots were stained with ORO and hematoxylin to visualize lesion area (black arrows) (A-E). Lesion area was reported as a fraction of the total aortic area (P). Aortic roots were stained with H&E to visualize necrotic areas (F-J). Necrotic areas were defined as acellular and are outlined in black. Necrotic area was reported as a percent of the lesion area (Q). Aortic roots were immunostained for CD68 (red) and nuclei (Hoechst; blue) to visualize macrophage-occupied area (K-O). Macrophage area was reported as the CD68^+^ percent of the lesion area (R). (S,T) Protein was collected from control (*n*=8), *Ripk3Δ^MΦ-Cre^* (*n*=5) and *Ripk3Δ^EC-Cre^* (*n*=4) aortas. Protein lysates were immunoblotted to identify CD11b and β-actin (loading control) (S) and quantified (T). For panels P,Q,R,T, each dot represents an individual animal. Statistics for panels P-R were calculated using one-way ANOVA. Overall ANOVA *P*-values (prior to the *post hoc* tests) are 0.42 (P), 0.01 (Q) and 0.30 (R). Despite the significant overall ANOVA *P*-value for panel Q, Dunnett's multiple comparisons test shows no significant difference from control aortic roots. Statistics for panel T were calculated using two-way ANOVA with Dunnett's multiple comparisons test with an overall ANOVA *P*-value of 0.01. Data are mean±s.d. Scale bars: 200 µm.

We also observed that the bulk of the increased plaque burden in *Ripk3Δ^MΦ-Cre^* and *Ripk3Δ^EC-Cre^* mice was detected in the descending and abdominal regions of the aorta, which are typically athero-protected, as opposed to the athero-prone arch region (Fig. 3C-E). In addition, we saw no significant differences in lesion size, necrotic core size, macrophage area, smooth muscle cell area, collagen area or calcification in the athero-prone aortic roots within the arch region (Fig. 4A-R and Fig. S3). We also detected no increase in apoptosis in the aortic root, though apoptotic cells were infrequent enough that quantification could not be performed (data not shown).

Interestingly, western blot analysis of the different aortic regions (arch, descending and abdominal; as demarcated in Fig. 3C) showed a significant increase in CD11b signal in the arch region of *Ripk3Δ^EC-Cre^* aortas (Fig. 4S,T). As CD11b is a monocyte/macrophage receptor component that assists in leukocyte trafficking (Schittenhelm et al., 2017), these data suggest that there is an increase in macrophage number in the arch region of *Ripk3Δ^EC-Cre^* aortas that is not reflected in the anti-CD68 immunostained aortic roots (Fig. 4R) or *en face* analyses (Fig. 3E).

### Triglyceride, cholesterol, glucose, and blood cell counts are unchanged between the genotypes

To confirm that the changes in *Ripk3Δ^MΦ-Cre^* and *Ripk3Δ^EC-Cre^* mice were not due to a change in overall metabolism or blood content, we analyzed plasma triglyceride, cholesterol and glucose levels and found no difference between the genotypes (Fig. S4). We also found no difference in peripheral mononuclear cells and other blood cells (Fig. S5).

### The main role of RIPK3 in atherosclerosis is likely not to initiate necroptosis

**Fig. 5.**
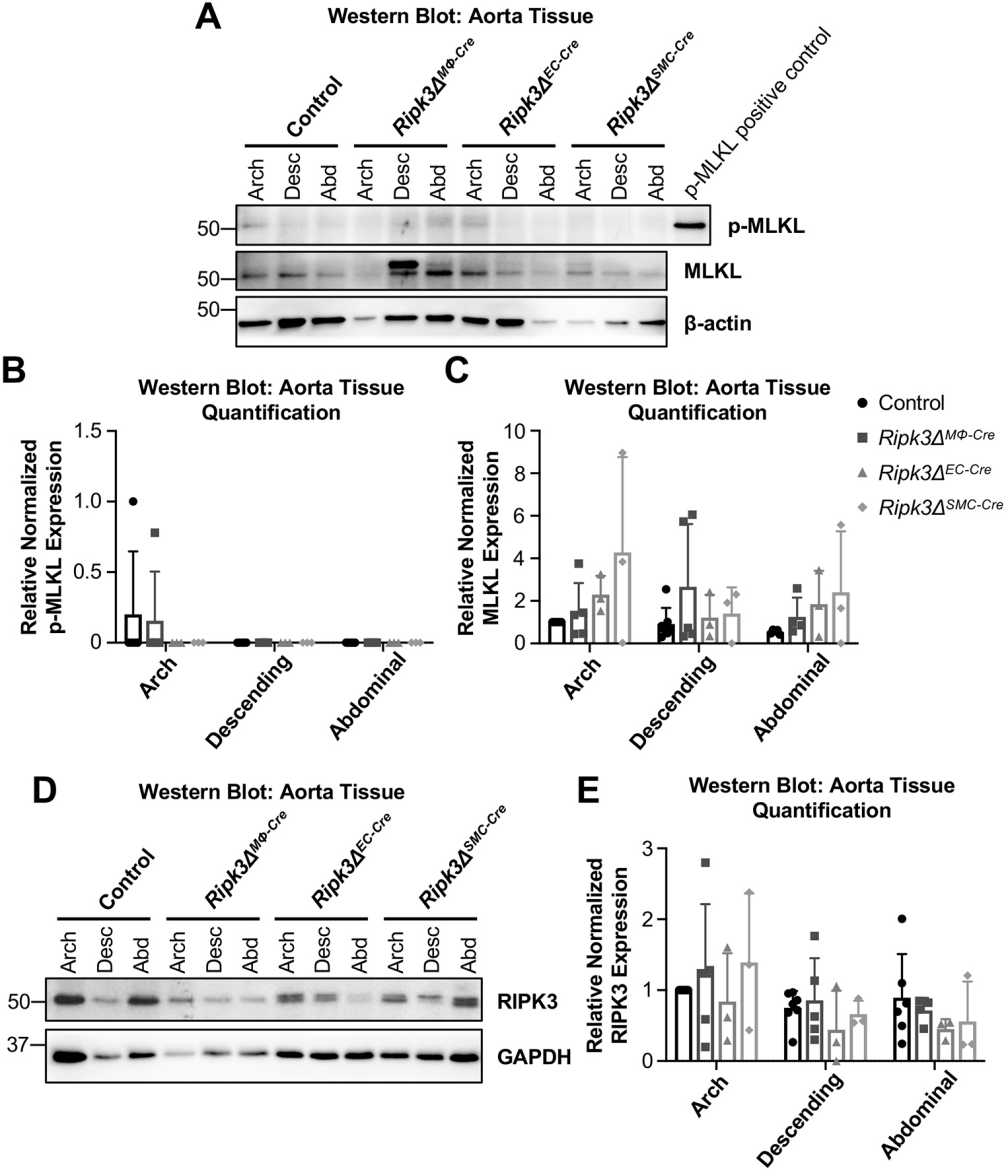
**p-MLKL levels are nearly undetectable in advanced plaques.** (A-C) After 3 months on a Western diet, protein was collected from control (*n*=7), *Ripk3Δ^MΦ-Cre^* (*n*=5), *Ripk3Δ^EC-Cre^* (*n*=3) and *Ripk3Δ^SMC-Cre^* (*n*=3) aortas. Protein lysates were immunoblotted to identify p-MLKL (Abcam; #ab196436), MLKL and β-actin (loading control) (A) and quantified (B,C). A faint positive p-MLKL signal can be seen in the representative blot in the control arch region; however, we could only detect p-MLKL in two out of the 18 aortas analyzed (B). Note that the same transfer membrane used for detecting CD11b (in Fig. 4S) was reprobed for p-MLKL and MLKL in A; the β-actin control blots are therefore the same in both figures. (D,E) Protein lysates were immunoblotted to identify RIPK3 and GAPDH (loading control) (D) and quantified (E). For panels B,C,E, each dot represents an individual animal. Statistics were calculated using two-way ANOVA. Overall ANOVA *P*-values are 0.76 (B), 0.07 (C) and 0.46 (E). Data are mean±s.d.

Owing to the unexpected exacerbation of atherosclerosis in *Ripk3Δ^MΦ-Cre^* and *Ripk3Δ^EC-Cre^* mice, we questioned whether necroptosis – which is considered to be inherently inflammatory and should increase atherosclerosis severity – was detectable in our atherosclerotic aortas. Because of widespread non-specific staining that occurred with the commercial antibodies that we used to immunostain for phosphorylated MLKL (p-MLKL), we alternatively used western blot techniques to detect this marker of necroptosis. We found that p-MLKL levels were so low as to be mostly undetectable by western blotting using two different anti-p-MLKL antibodies (Fig. 5A-C and Fig. S6). Thus, we suspect that the main role of RIPK3 in atherosclerosis is not to cause macrophage necroptosis, but rather to prevent macrophages and endothelial cells from becoming pro-inflammatory.

Furthermore, we found that RIPK3 protein levels in different aortic regions and genotypes were not significantly different from control levels by western blot (Fig. 5D,E). If macrophages, smooth muscle cells or endothelial cells predominantly expressed RIPK3 over the other cell types, then we would have expected to see a decrease in RIPK3 expression in the corresponding mutant genotype. As we did not, this suggests that the bulk of RIPK3 expression detected by western blotting in the aorta is not restricted to any one of these three cell types.

### *Ripk3* deficiency impacts MCP-1 and IL-1β expression in BMDMs, and MCP-1 and E-selectin expression in cultured endothelial cells

**Fig. 6.**
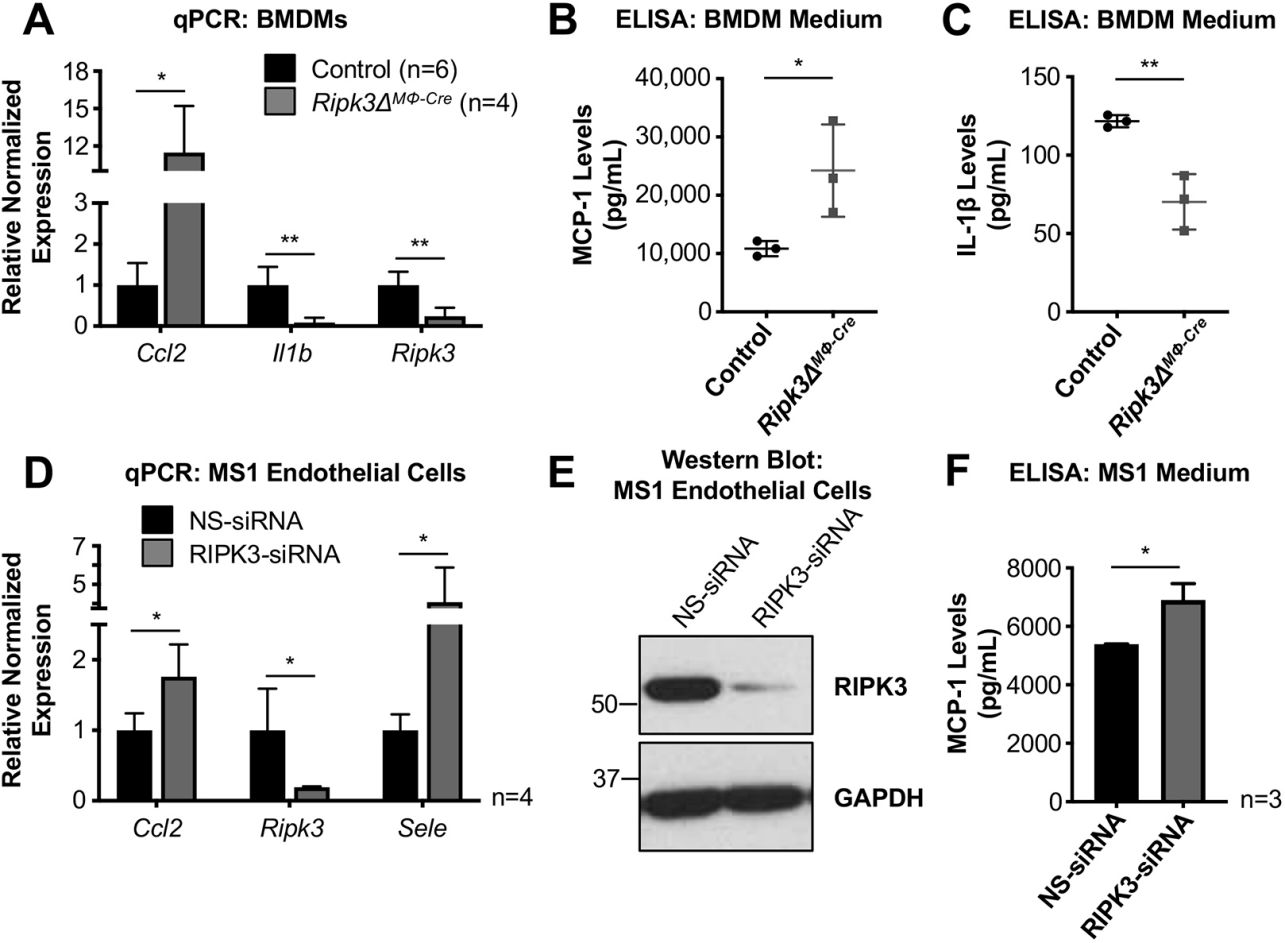
***Ripk3-*deficiency impacts MCP-1 and IL-1β expression in BMDMs, and MCP-1 and E-selectin expression in cultured endothelial cells.** (A-C) BMDMs were isolated from control (*n*=6) and *Ripk3Δ^MΦ-Cre^* (*n*=4) bone marrow, and RNA and medium were collected 7 days later. RNA was converted to cDNA and analyzed by qPCR (A). ELISA assays for MCP-1 (B) and IL-1β (C) were performed on the BMDM medium. Each dot (*n* value) represents a BMDM isolation from an individual animal. (D-F) MS1 endothelial cells were transfected with NS- and RIPK3-siRNA oligos for 24 h and were cultured in low-serum medium for another 24 h before RNA, medium and protein were collected (*n*=4 independent experiments). RNA was converted to cDNA and analyzed by qPCR (D). Protein lysates were immunoblotted for RIPK3 and the loading control, GAPDH (*n*=4 independent experiments) (E). An ELISA assay for MCP-1 was performed on the BMDM medium (*n*=3 independent experiments) (F). Statistics were calculated using unpaired *t*-tests, with Welch's correction when necessary. *P<0.05, **P<0.01. Data are mean±s.d.

To try to understand how *Ripk3* deletion in macrophages could lead to an increase in atherosclerosis severity, we isolated and cultured BMDMs from control and *Ripk3Δ^MΦ-Cre^* bone marrow and performed real-time quantitative PCR (qPCR) for a panel of genes that are related to necroptosis, inflammation, macrophage activation and endothelial cell activation (Fig. 6A and Fig. S7A). We found that *Ccl2* (MCP-1) and *Il1b* (IL-1β) transcripts were significantly changed in *Ripk3Δ^MΦ-Cre^* BMDMs. *Ccl2* was upregulated almost 12-fold in *Ripk3Δ^MΦ-Cre^* BMDMs over control BMDMs, and ELISA analysis of the medium confirmed the increase in MCP-1 and decrease in IL-1β (Fig. 6B,C). RIPK3 has already been implicated in IL-1β processing (Weinlich et al., 2016) and has been shown to promote *Il1b* transcription (Moriwaki et al., 2017), but this is the first report implicating RIPK3 in the suppression of *Ccl2* expression.

To try to understand how *Ripk3* deletion in endothelial cells could lead to an increase in atherosclerosis severity, we utilized a cultured murine pancreatic endothelial cell line (MS1 cell line), which we chose for this study because it expresses high levels of endogenous RIPK3. By knocking down RIPK3 using siRNA oligos and looking at a similar panel of genes, we found that *Ccl2* transcripts and MCP-1 protein levels were also elevated in endothelial cells, although not to the extent seen in BMDMs (Fig. 6D-F and Fig. S7B). Furthermore, *Sele* (E-selectin) transcripts were upregulated approximately fourfold in RIPK3-knockdown MS1 cells. This is relevant as E-selectin is a glycoprotein surface receptor on endothelial cells that assists in the recruitment of circulating leukocytes (McEver, 2015).

Based on these data, we hypothesized that increased macrophage MCP-1 levels contributed to the more severe atherosclerosis we detected in *Ripk3Δ^MΦ-Cre^* mice, as macrophage-specific expression of MCP-1 is known to exacerbate atherosclerosis (Aiello et al., 1999). We also hypothesized that increased endothelial MCP-1 and E-selectin levels contributed to the elevated atherosclerosis severity in *Ripk3Δ^EC-Cre^* mice, as these molecules would increase the recruitment of leukocytes and therefore accelerate atherosclerosis.

### MCP-1 levels are elevated in *Ripk3Δ^MΦ-Cre^* abdominal aortas, and E-selectin levels trend higher in *Ripk3Δ^EC-Cre^* aortas

**Fig. 7.**
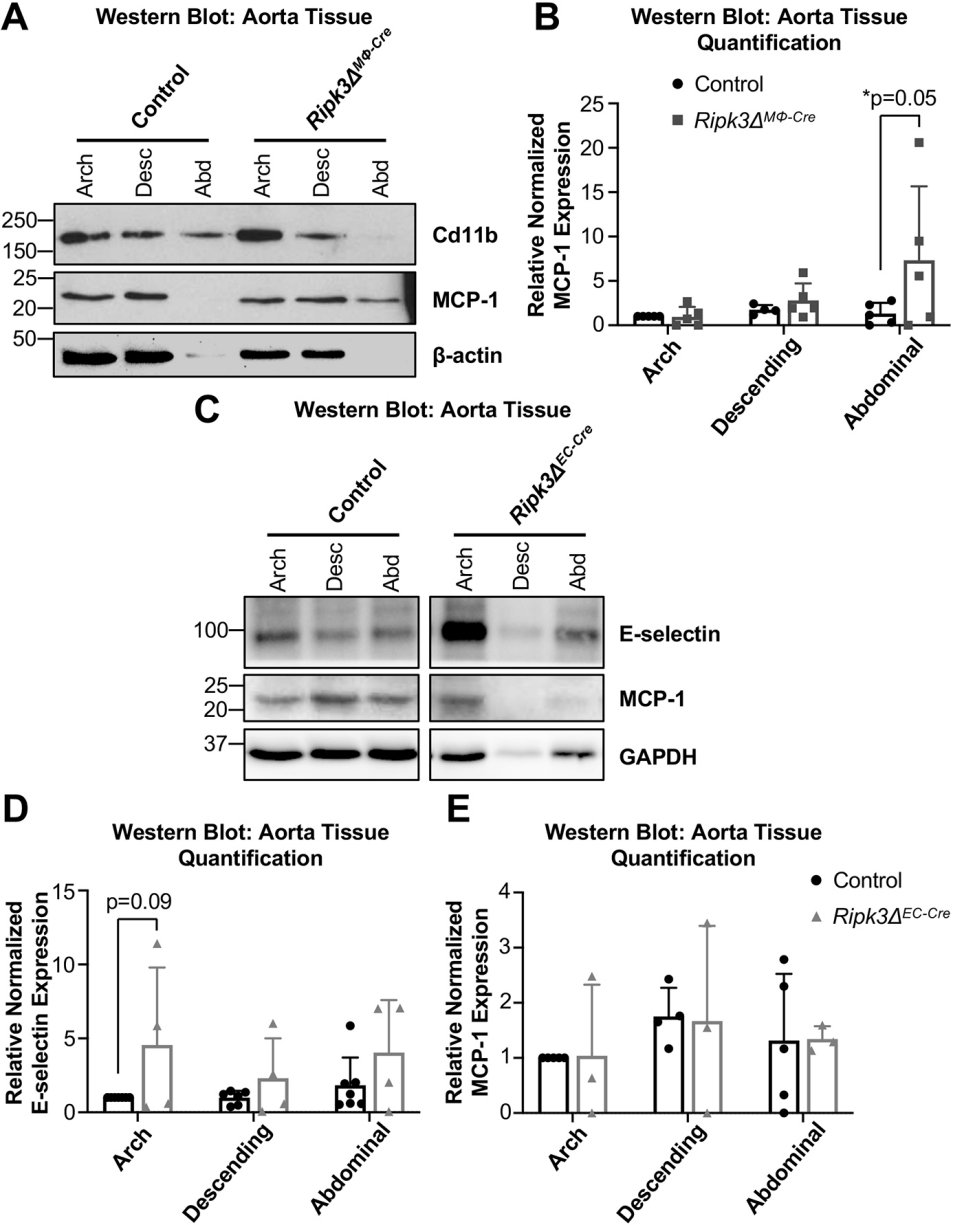
**MCP-1 levels are elevated in *Ripk3Δ^MΦ-Cre^* abdominal aortas, and E-selectin levels trend higher in *Ripk3Δ^EC-Cre^* aortas.** (A-E) After 3 months on a Western diet, protein was collected from control (*n*=8), *Ripk3Δ^MΦ-Cre^* (*n*=5) and *Ripk3Δ^EC-Cre^* (*n*=4) aortas. Protein lysates were immunoblotted to identify MCP-1, E-selectin, GAPDH and β-actin (loading controls) (A,C) and quantified (B,D,E). The immunoblot in panel C shows non-contiguous lanes of a single gel. For panels B,D,E, each dot represents an individual animal. Statistics were calculated using two-way ANOVA, and Sidak's multiple comparisons test in panel B. Overall ANOVA *P*-values (prior to the *post hoc* tests) are 0.09 (B), 0.02 (D) and 0.99 (E). Data are mean±s.d.

To see whether the BMDM and MS1 cell functions of RIPK3 translated to our *in vivo* tissues, we analyzed MCP-1 and E-selectin levels in the arch, descending and abdominal regions of mouse aortas using western blotting. *Ripk3Δ^MΦ-Cre^* aortas exhibited an increase in MCP-1 in the abdominal region that was robust, if variable, compared to controls (Fig. 7A,B). This supports the hypothesis that loss of RIPK3 in macrophages increases macrophage MCP-1 levels in the abdominal region of *Ripk3Δ^MΦ-Cre^* aortas, which correlates with the increase in abdominal lesion area (Fig. 3D).

In contrast, *Ripk3Δ^EC-Cre^* aortas did not show an increase in MCP-1 but showed an upward trend in E-selectin levels by western blot (Fig. 7C,D). We immunostained regions of the descending aorta to see whether the increase in E-selectin would be more apparent using another technique; however, we did not see an increase in E-selectin intensity (Fig. S8A,B), though this could be attributed to the fact that the E-selectin signal was already very high in control aortas and thus it was difficult to see a change in intensity. We also examined ICAM1 and VCAM1 levels because of their high expression in athero-prone regions and their connection to inflammation (Nakashima et al., 1998). However, neither gene was altered in *Ripk3-*deficient BMDMs or MS1 cells (Fig. S7); nor were ICAM1 and VCAM1 levels significantly different in *Ripk3Δ^EC-Cre^* or *Ripk3Δ^MΦ-Cre^* aortas (Fig. S8C-E).

In addition, as RIPK1 is a homologous kinase upstream of RIPK3 and is often tied to RIPK3 function (Moriwaki and Chan, 2016), we immunoblotted for RIPK1 in the various regions of the aorta. When *Ripk3* is deleted in a cell-specific manner, RIPK1 levels are not noticeably altered in the aorta lysate (Fig. S9A). However, aortas with heterozygous and global deletion of *Ripk3* show an appreciable decrease in RIPK1 levels (Fig. S9B), indicating that RIPK1 protein levels are tied to RIPK3 levels. However, owing to the fact that RIPK1 plays roles in regulating necroptosis, apoptosis, autophagy, inflammation and cell survival pathways (Lin, 2014), it is difficult to speculate how the substantial decrease in RIPK1 affects atherosclerosis in *Ripk3*-deficient animals.

### Plasma IL-1β levels are increased in *Ripk3Δ^Global^* mice, whereas plasma MCP-1 levels are unaffected by *Ripk3* deletion

**Fig. 8.**
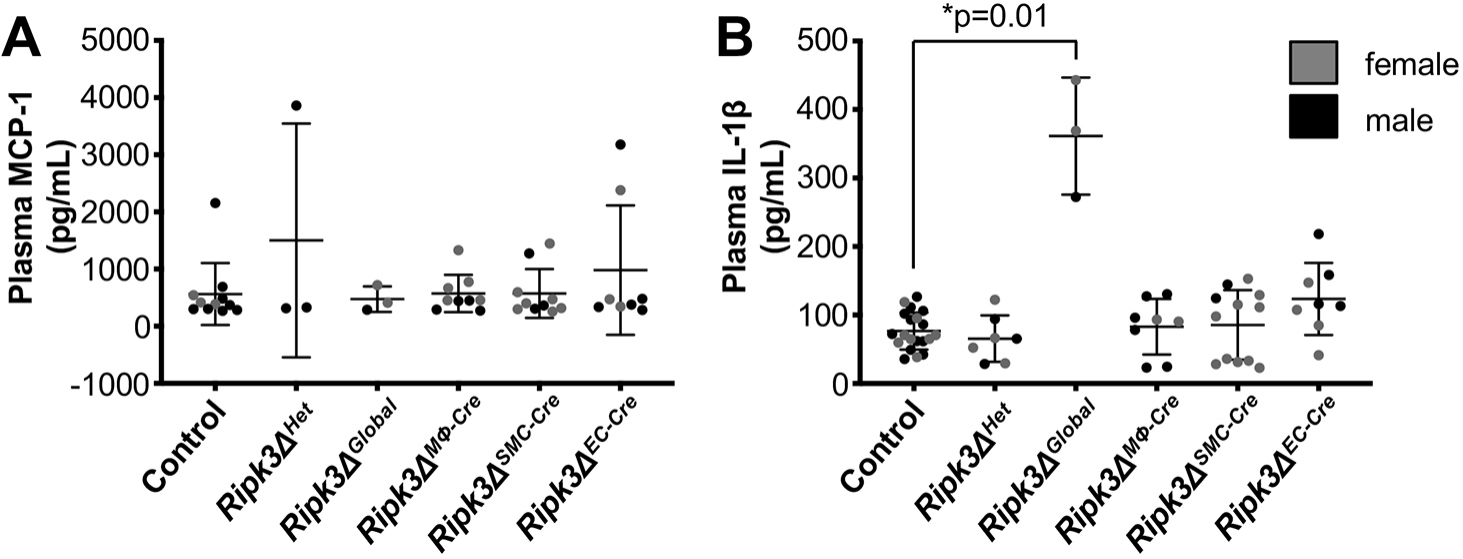
**Plasma IL-1β levels are increased in *Ripk3Δ^Global^* mice, whereas plasma MCP-1 levels are unaffected by *Ripk3* deletion.** (A,B) After mice were fed a Western diet for 3 months, plasma was collected and analyzed by ELISA for MCP-1 (A) and IL-1β (B). Each dot represents an individual animal. Statistics for both panels were calculated using Kruskal–Wallis tests, and Dunn's multiple comparisons test for panel B. Overall ANOVA *P*-values (prior to the *post hoc* tests) are 0.97 (A) and 0.01 (B). Data are mean±s.d.

To determine whether the *in vitro* changes we detected in MCP-1 or IL-1β levels could be seen in the plasma of our mutant mice, we performed an ELISA assay on the plasma samples collected from the different genotypes. However, we detected no differences in MCP-1 levels (Fig. 8A). Although *Ripk3Δ^MΦ-Cre^* plasma did not show a decrease in IL-1β levels as would have been expected from the BMDM data, we did see a significant increase of IL-1β in the *Ripk3Δ^Global^* plasma (Fig. 8B). This suggests that, although RIPK3 has been reported to positively regulate IL-1β processing (Weinlich et al., 2016), it may also play a suppressive role in certain conditions or cell types.

## DISCUSSION

Until now, RIPK3 has been associated primarily with necroptosis and – to a lesser extent – other pro-inflammatory pathways separable from necroptosis. In this study, which is the first to explore the cell-specific function of RIPK3 in the atherosclerotic aorta, we demonstrate that RIPK3 plays an unexpected athero-protective role in macrophages and endothelial cells. Here, we propose that the main role of RIPK3 in atherosclerosis is neither to promote necroptosis nor IL-1β processing, which are the previously described functions for RIPK3 (Weinlich et al., 2016). Instead, our data indicate that RIPK3 may play an anti-inflammatory role by suppressing MCP-1 expression in macrophages and E-selectin expression in endothelial cells (data summarized in Fig. S10). These results provide novel information about RIPK3 in an inflammatory vascular disease and raise questions about the efficacy and safety of targeting it in a clinical setting.

We were surprised by the fact that our mice with global and heterozygous *Ripk3* deletions did not resemble previously reported *Ripk3* mutant mice that showed a dose dependent improvement in atherosclerotic lesion area (Lin et al., 2013). It is possible that this inconsistency may be due to different background strains in the mice that were analyzed. The *Ripk3^−/−^* mice used in the original study were derived from C57BL/6N embryonic stem cells (Newton et al., 2004), whereas our *Ripk3^fl/fl^* mice were derived from C57BL/6J embryonic stem cells (Colijn et al., 2019). Lin et al. reported that they performed their study on a C57BL/6J background; however, it is unclear for how many generations the original C57BL/6N strain was crossed onto the C57BL/6J strain. Although this distinction may appear to be minor, much work has been done to map the genetic and metabolic differences between the two backgrounds, and a difference of phenotype is entirely possible (Fontaine and Davis, 2016). Thus, in addition to revealing cell-specific roles for RIPK3, we may also have uncovered a background-specific regulation or function of RIPK3 that future studies should consider.

Another potential cause of the discordant results from our genetic study and that of Lin et al. is the different environments in which the mice were housed and analyzed. The two studies were performed at different institutions and with different suppliers of the Western diet (Lin et al., 2013). This raises questions about the contribution of microbiota to RIPK3-dependent functions within the context of atherosclerosis. It has already been well established that the microbiome can influence atherosclerosis (Jie et al., 2017), thus microbiota that differentially affect inflammatory pathways could also differentially affect RIPK3 activation. As humans have such diverse and dissimilar microbiota, the effect of the microbiome on RIPK3 function should be explored before RIPK3 is considered as a therapeutic target.

It is also possible that differences between the *Ripk3^−/−^* genetic model used by Lin et al. and our *Ripk3^fl/fl^* genetic model could contribute to the discrepancies between our studies. Specifically, whereas our *Ripk3^fl/fl^* mouse model results in removal of exons one through nine of *Ripk3* upon Cre-mediated recombination, only the first three exons of *Ripk3* are targeted for deletion in the *Ripk3^−/−^* mouse model (Colijn et al., 2019; Newton et al., 2004). *Ripk3* has been reported to have two alternative splicing variants in addition to the full length form (Yang et al., 2005), and although part of the alternative spliced sequences include the first three exons and therefore may not be transcribed in the *Ripk3^−/−^* mouse, this was not addressed in the design of the *Ripk3* knockout locus. Though unlikely, it is possible that a truncated or frame-shifted form of RIPK3 was present in the mice used by Lin et al. that could account for the difference between our results.

Finally, it is worth noting that another study that looked at atherosclerosis in an *Apoe^−/−^;Ripk3^−/−^* mouse model did not completely replicate the study by Lin et al., although it did conclude that RIPK3-mediated necroptosis contributes to atherosclerosis pathology (Meng et al., 2015). The authors reported no difference in macrophage infiltration and did not observe a significant difference in aortic root lesion area in very advanced plaques, which is contrary to the study by Lin et al. Though Meng et al. did not bring attention to these differences, it is clear that there is some variation in RIPK3 atherosclerosis studies that should be considered.

Surprisingly, despite detecting notable atherosclerotic phenotypes associated with *Ripk3* deficiency, we were unable to detect much *Ripk3* transcript expression in atherosclerotic plaques. Although the commercial RIPK3 antibodies we used were unsuitable for assessing patterns of RIPK3 expression in plaque tissues by immunostaining, we were able to determine by immunoblotting that neither macrophages, smooth muscle cells, nor endothelial cells are the main sources of RIPK3 expression in plaques, and that RIPK3 protein expression is not restricted to a particular region of the aorta. Nevertheless, the limitations in RIPK3 immunostaining prevented us from assessing Cre specificities and efficiencies directly in plaque tissues, which would have been ideal. As plaque tissue is difficult to immunostain owing to high levels of non-specific background signal, we propose that the best way to determine RIPK3 protein expression patterns accurately in the context of atherosclerosis would be to use a RIPK3-tagged reporter mouse, such as the RIPK3-GFP mouse developed by Moriwaki et al. (2017).

A major finding from our study is that RIPK3 plays unexpected anti-inflammatory roles in atherosclerosis and contributes to the athero-protection of the descending and abdominal aortic regions. However, our study is not the first to indicate that RIPK3 activity can lead to the suppression of inflammation. One report demonstrates that promoting RIPK3 activation in cultured mouse fibroblasts – through TNF-treatment and caspase inhibition – results in a marked decrease in secretion of inflammatory cytokines (Kearney et al., 2015). Our findings that IL-1β is elevated in plasma from *Ripk3Δ^Global^* mice (Fig. 8B) and that MCP-1 is upregulated in the abdominal aortic region of *Ripk3Δ^MΦ-Cre^* mice (Fig. 7B) provide *in vivo* evidence of anti-inflammatory roles for RIPK3. In addition, the upward trends in E-selectin (Fig. 7D) and in CD11b (Fig. 4T) expression in *Ripk3Δ^EC-Cre^* aortas provide further indications that RIPK3 activity suppresses inflammation in a cell-type specific manner during atherosclerosis. Intriguingly, the marked elevation in pro-inflammatory IL-1β levels from *Ripk3Δ^Global^* plasma did not track with an increase in plaque severity. In fact, aortic plaques were less severe in our *Ripk3Δ^Global^* mice than in our *Ripk3Δ^EC-Cre^* and *Ripk3Δ^MΦ-Cre^* animals. Ultimately, we do not know the reason for the phenotypic differences between our mice with global and cell-specific *Ripk3* deletion, but our findings indicate that RIPK3 may play opposing roles in different interacting cell types and that global deletion may negate the damaging effects of cell-specific RIPK3 deletion in the context of atherosclerosis.

One important question that arises from our findings is whether RIPK3-mediated necroptosis suppresses inflammation during atherosclerosis progression, as has been suggested for the cultured mouse fibroblast study cited above (Kearney et al., 2015). It has been proposed that *Mlkl* deletion is a more definitive genetic approach than *Ripk3* deletion for determining how necroptosis influences various pathologies, as RIPK3 has roles outside of necroptosis and MLKL is downstream of RIPK3 in the necroptosis pathway (Newton et al., 2016). As one published abstract hints that *Mlkl*-deficiency does not improve atherosclerosis (Rasheed et al., 2018), and as we were unable to detect p-MLKL in the atherosclerotic plaques we analyzed (Fig. 5A,B and Fig. S6), we propose that necroptosis contributes minimally to atherosclerosis progression and to the anti-inflammatory roles we have defined for RIPK3 in this study.

Altogether, we believe this study challenges the field to reevaluate the prevalent idea that RIPK3 promotes pathologies exclusively through necroptotic or other pro-inflammatory mechanisms. Our data indicate that RIPK3 has cell-specific functions and that some of those may be beneficial and anti-inflammatory in the context of the vasculature. Exploring other protective roles for RIPK3 will help clarify the potential risks and benefits of targeting RIPK3 therapeutically in various diseases.

## MATERIALS AND METHODS

### Mice (*Mus musculus*)

*Apoe^−/−^* mice on the C57BL/6J background (Piedrahita et al., 1992), *SM22-Cre* transgenic mice (Holtwick et al., 2002), *LysM-Cre* transgenic mice (Clausen et al., 1999), *VE-Cadherin-Cre* transgenic mice (Alva et al., 2006) and *Ripk3-*floxed (*Ripk3^fl/fl^*) mice on the C57BL/6J background (Colijn et al., 2019) have been described. *Ripk3-*knockout (*Ripk3^Δ/Δ^*) mice were generated by utilizing the germ-line excision phenomenon that occurs when Cre activity aberrantly turns on in the gonads of the breeders (Harno et al., 2013), thereby altering the *Ripk3*-floxed allele into a *Ripk3-*knockout allele. Mice were maintained and interbred on the C57BL/6J background at the Oklahoma Medical Research Foundation (OMRF) animal facility. Male and female breeders were used interchangeably for many generations to ensure all target genotypes were on a highly uniform background strain for the analyses. The OMRF Institutional Animal Care and Use Committee approved all animal use protocols.

### Genotyping

*Apoe^−/−^*, *SM22-Cre*, *LysM-Cre*, *VE-Cadherin-Cre* and *Ripk3*-floxed mice were genotyped as previously described (Colijn et al., 2019; Holtwick et al., 2002; Goren et al., 2009; Mani et al., 2013; Wiley et al., 2015). *Ripk3-*knockout genotyping was performed by PCR using the forward primer 5′-CCATCCTCCCTTCATCAAAA-3′ and reverse primer 5′-GAATGCAAATGCAGGGTCTT-3′, which are located upstream of the 5′ LoxP site and downstream of the 3′ LoxP site, respectively. The PCR produced a 312-bp band to signify that *Ripk3* excision had occurred.

### Atherosclerosis induction

*Apoe^−/−^* mice were crossed with the *Ripk3^fl/fl^* line and either *SM22-Cre*, *LysM-Cre* or *VE-Cadherin-Cre* mice to generate the target genotypes: *Apoe^−/−^;Ripk3^fl/fl^;SM22-Cre* (*n*=18), *Apoe^−/−^;Ripk3^fl/fl^;LysM-Cre* (*n*=19), *Apoe^−/−^;Ripk3^fl/fl^;VE-Cadherin-Cre* (*n*=14), *Apoe^−/−^;Ripk3^fl/Δ^* (*n*=9), *Apoe^−/−^;Ripk3^Δ/Δ^* (*n*=9) and *Apoe^−/−^;Ripk3^fl/fl^* Cre-negative littermates as controls (*n*=36). Two-month-old male and female mice were fed a Western diet (21.2% fat, 0.2% cholesterol; TD.88137, Envigo) for three months to induce atherosclerosis progression. The mice were then euthanized. A cardiac puncture was performed to collect blood. Mice were then perfused with PBS followed by 4% paraformaldehyde (PFA). Aortas were cleaned of fat and removed along with the hearts. Bone marrow was collected from the femur and tibia as described (Amend et al., 2016).

### *En face* analysis

The aorta was cut open longitudinally from the iliac arteries to the aortic root and pinned open on parafilm. After further fixation with 4% PFA, lesions were visualized with an Oil Red O stain (ORO, Newcomer Supply), and residual fat was removed. Individual high-resolution images were stitched together by hand to create the entire *en face* aorta images. Aortic images were further analyzed with NIS-Elements software for ORO staining, and data are reported as the percentage of the aortic surface covered by lesions.

### Aortic root analysis

Hearts were fixed in 1% PFA overnight, placed in 20% sucrose, then incubated in a 1:1 mixture of 20% sucrose and Optimal Cutting Temperature compound (OCT; Sakura Finetek) overnight and frozen in OCT the next day. Whole aortic roots were sectioned at 10 µm with a Microm HM 505 E cryotome (Microm International) and adhered to Denville UltraClear Microscope Slides (Denville Scientific). Slides were frozen until further use. All analyses were performed using NIS-Elements software.

#### Lesion area

Aortic roots were stained with ORO to identify lesion area. Slides were first immersed in water, then 60% isopropanol for 30 s, ORO for 30 min, then 60% isopropanol for 30 s to rinse. Slides were co-stained with hematoxylin, mounted with 1,4-diazabicyclo[2.2.2]octane (DABCO) and coverslipped. Six aortic root sections per mouse were analyzed at 60 μm apart. The average lesion area from each mouse was normalized to the average total aortic area and reported as a fraction.

#### Necrotic area

Aortic roots were stained with hematoxylin and eosin (H&E) for necrotic core quantification. Three aortic root sections per mouse were analyzed at 120 μm apart. Necrotic cores were identified as acellular areas (negative for H&E). The average necrotic area was reported as the percent necrotic area of the total lesion area.

#### Macrophage and smooth muscle cell area

Macrophages and smooth muscle cells of the aortic root were visualized using rat anti-CD68 (1:250; #MCA1957; Bio-Rad) and Cy3-conjugated anti-aSMA (1:250; #C6198; Sigma-Aldrich) antibodies, respectively. Sections were blocked with 5% bovine serum albumin (BSA) for 2 h, incubated with primary antibody overnight, and incubated with secondary antibody Cy3 donkey anti-rat IgG (1:500; Jackson ImmunoResearch) for CD68 and Hoechst for nuclei (20 µg/ml) for 1 h. For macrophages, three aortic root sections per mouse were analyzed at 120 μm apart. For smooth muscle cells, one aortic root section per mouse was analyzed from the middle of the root. The average CD68^+^ area or aSMA^+^ area was reported as the percent area of the total lesion area.

#### Collagen deposition

Aortic roots were stained with the Picrosirius Red Stain Kit (Polysciences) for collagen area quantification. Sections were stained with Solution A for 2 min, Solution B for 1 h and Solution C for 2 min. Two aortic root sections per mouse were analyzed at 180 μm apart. Collagen was identified as the green, yellow and red colors that can be visualized under polarized light. The average collagen area was reported as the percent collagen area of the total lesion area.

#### Calcification

Aortic roots were stained with Alizarin Red S (Sigma-Aldrich) to detect calcification. Sections were stained with a 1% Alizarin Red S (pH 4.2) solution for 5 min. Two aortic root sections were analyzed at 180 μm apart. Calcification was recorded as either present or absent.

### Plasma triglyceride, cholesterol and glucose analysis

After blood was drawn by cardiac puncture, plasma was isolated using Microvette CB 300 LH tubes (Sarstedt). The concentrations of total cholesterol, LDL/VLDL cholesterol, HDL cholesterol and triglycerides were determined using enzymatic colorimetric assays (#E2HL-100 and #ETGA-200; BioAssay Systems) as per the manufacturer's instructions. Glucose levels were detected using the Contour^®^Next EZ Blood Glucose Monitoring System with Contour^®^Next test strips.

### Complete blood count

Blood was collected into Microvette 500 K3E tubes (Sarstedt). Complete blood counts were measured with a Hemavet 950 veterinary hematology analyzer (Drew Scientific).

### RNA *in situ* hybridization

Hearts were fixed in 4% PFA for 24 h and passed through a sucrose gradient as per RNAScope^®^ protocol, then embedded in OCT. After 10 μm aortic root sections were adhered to slides coated with Poly-L-Lysine (#P4707; Sigma-Aldrich), slides were then frozen until use. RNA *in situ* hybridization was performed using the RNAScope^®^ Fluorescent Multiplex Reagent Kit (#320850; Advanced Cell Diagnostics). RNA retrieval was performed first by boiling the slides for 6 min in the Target Retrieval Buffer (#322000; Advanced Cell Diagnostics) and then 1 h with Protease III at 40°C. RNAScope^®^ probe hybridization protocol was followed for the *Ripk3* probe (#462541-C3; Advanced Cell Diagnostics). Positive control probes for *Ubc* and *Polr2a* (#320881; Advanced Cell Diagnostics) were used for each sample. After mounting with Prolong Gold (Thermo Fisher Scientific), images were obtained with a Nikon Eclipse Ti-E microscope.

### Immunofluorescence in tissue sections

Hearts were fixed in 1% PFA overnight, placed in 20% sucrose, then incubated in a 1:1 mixture of 20% sucrose and OCT overnight and frozen in OCT the next day. Sections were cut at 10 µm and frozen until use. Sections were permeabilized in 0.1% Triton X-100 in PBS for 15 min then blocked in 5% BSA for 2 h at room temperature. Sections were incubated in primary antibody in 1% BSA overnight at 4°C, washed three times quickly in ice-cold 1% BSA, then incubated for 1 h at room temperature with secondary antibody in 1% BSA with Hoechst (20 µg/ml). Sections were quickly washed three times with cold 1% BSA and coverslipped with DABCO. Images were obtained using a Nikon Eclipse Ti-E microscope with NIS-Elements software. Primary antibodies used for immunofluorescence were: goat anti-PECAM-1 (1:100; #AF3628; R&D Systems), rat anti-CD68 (1:250; #MCA1957; Bio-Rad), Cy3-conjugated anti-aSMA (1:250; #C6198; Sigma-Aldrich) and chicken anti-E-selectin (1:100; #AF575; R&D Systems). Secondary antibodies used were: Cy3 donkey anti-rat IgG (#712-165-153), FITC donkey anti-goat IgG (#705-095-003) and Alexa 594 donkey anti-chicken IgY (#703-585-155). All secondary antibodies were acquired from Jackson ImmunoResearch and used at a 1:500 dilution.

### Isolation and differentiation of BMDMs

BMDMs were isolated from tibias and femurs of *Apoe^−/−^;Ripk3^fl/fl^*, *Apoe^−/−^;Ripk3^fl/fl^;LysM-Cre*, *Apoe^−/−^;Ripk3^fl/fl^;VE-Cadherin-Cre* or *Apoe^−/−^;Ripk3^Δ/Δ^* mice and differentiated into macrophages using DMEM (#12-604F; Lonza) supplemented with 10% fetal bovine serum (FBS; HyClone), 1× antibiotic-antimycotic (#15240-062; Gibco), 2 mM L-glutamine (#95057-448; HiMedia), 1 mM sodium pyruvate (#25-000-Cl; Mediatech), 1× MEM non-essential amino acids (#11140-050; Gibco) and rhM-CSF (20 ng/ml; #78057; Stemcell Technologies) for 7 days. Differentiation was confirmed by a phagocytosis assay and by immunocytochemistry for the macrophage markers CD11b and CD68. For the phagocytosis assay, BMDMs were treated with FluoSpheres polystyrene microspheres (#F13082; Thermo Fisher Scientific) for 4 h, co-stained with Hoechst (10 µg/ml), washed, fixed and then visualized on a Nikon Eclipse Ti-E microscope. Phagocytic cells were identified as TRITC^+^ and quantified.

### Immunocytochemistry

Bone marrow was plated on chamber slides (Lab-Tek) and differentiated into BMDMs as described above. On day 7, slides were fixed with 4% PFA for 10 min, permeabilized with 0.5% saponin for 10 min, blocked [1% BSA, 22.52 mg/ml glycine in PBS with 0.1% Tween 20 (PBST)] for 1 h and incubated in primary antibody diluted in 1% BSA in PBST overnight at 4°C. After washing with PBS, slides were incubated in secondary antibody with Hoechst (20 µg/ml) in 1% BSA in PBST for 1 h. After mounting with DABCO, images were obtained with a Nikon Eclipse Ti-E microscope. Primary antibodies used for immunocytochemistry were: rabbit anti-CD11b (1:250; #EPR1344; Abcam) and rat anti-CD68 (1:250; #MCA1957; BioRad). Secondary antibodies used were acquired from Jackson ImmunoResearch and used at a 1:500 dilution: Cy3 donkey anti-rat IgG (#712-165-153) and Cy5 donkey anti-rabbit IgG (#711-175-152).

### Cell culture and treatment

The murine MS1 pancreas-derived endothelial cell line (#CRL-2279; ATCC) was maintained in DMEM supplemented with 5% FBS (HyClone) and 1× antibiotic-antimycotic (#15240-062; Gibco). MS-1 cells were tested quarterly for mycoplasma contamination by PCR. For RIPK3 knockdown, cells were treated with RNAiMAX transfection reagent (Thermo Fisher Scientific) and 50 nM of RIPK3 Silencer Select or non-targeting control siRNA oligonucleotides (#s80756 and #4390844, respectively; Ambion) in serum-free OptiMEM (Invitrogen). After 24 h, medium was replaced with low-serum medium. After another 24 h, cells and medium were harvested for subsequent transcript or protein analyses. On day 7 of BMDM differentiation, BMDMs and medium from either *Apoe^−/−^;Ripk3^fl/fl^* or *Apoe^−/−^;Ripk3^fl/fl^;LysM-Cre* mice were harvested for subsequent transcript or protein analyses.

### Western blot assays

MS1 cells and BMDMs were lysed in RIPA buffer [50 mM Tris-HCl (pH 7.4), 150 mM NaCl, 1% NP-40, 0.1% SDS, 1 mM EDTA, 0.5% sodium deoxycholate] with Protease Inhibitor Cocktail (#78430; Thermo Fisher Scientific) and Phosphatase Inhibitor Cocktail (#78420; Thermo Fisher Scientific). Aorta tissues were flash frozen and ground with a mortar and pestle then resuspended in RIPA buffer with the protease and phosphatase inhibitors. Protein concentration was determined using the Pierce BCA Protein Assay Kit (#23227, Thermo Fisher Scientific) and a NanoDrop 2000 from Thermo Fisher Scientific. Then, 5-10 μg protein was electrophoresed on a 12% SDS-PAGE gel, then transferred to a PVDF membrane, and then blocked in 5% nonfat dry milk in Tris-buffered saline with 0.1% Tween 20 (TBST) for 1 h. Membranes were cut into sections based on target protein molecular weights to optimize the number of proteins that could be detected. Primary antibodies (diluted in TBST for detection of phosphorylated proteins or 5% milk-TBST for all other proteins) were incubated at 4°C overnight with gentle agitation, and membranes were then washed three times (10 min each) in TBST. HRP-conjugated secondary antibodies (diluted in 5% milk-TBST) were applied at room temperature for 1 h with gentle agitation, and membranes were then washed four times (15 min each) in TBST. Secondary antibodies were detected using ECL Western Blotting Detection Reagent (#34076 or #34096; Thermo Fisher Scientific). Membranes were then stripped using Restore™ PLUS Western Blot Stripping Buffer (#46430; Thermo Fisher Scientific) and re-blotted with primary antibodies raised in a different species to prevent residual HRP-signal. Densitometry analysis was performed using ImageJ software. Primary antibodies used for immunoblotting were: goat anti-ICAM1 (1:5000; #AF796; R&D Systems), goat anti-VCAM1 (1:1000; #AF643; R&D Systems), rabbit anti-CD11b (1:3000; #EPR1344; Abcam), chicken anti-E-selectin (1:1000; #AF575; R&D Systems), rabbit anti-RIPK3 (1:4000; #NBP1-77299; NOVUS Biologicals), goat anti-MCP-1 (1:2000; #AF479; R&D Systems), rabbit anti-MLKL (1:1000; #orb32399; Biorbyt), rabbit anti-phosphorylated-MLKL (1:1000; #ab196436; Abcam), rabbit anti-phosphorylated-MLKL (1:1000; #37333; Cell Signaling Technology), rabbit anti-RIPK1 (1:1000; #ab202985; Abcam), rabbit anti-β-actin (1:10,000; #4970; Cell Signaling Technology) and rabbit-anti-GAPDH (1:20,000, #G9545; Sigma-Aldrich).

### qPCR assays

To analyze transcript levels, total RNA isolated from MS1 endothelial cells or BMDMs was purified and treated with RNase-free DNaseI (Qiagen). cDNA was prepared using the iSCRIPT™ Reverse Transcriptase Kit (Bio-Rad), and qPCR was performed using 2X SYBR green qPCR master mix (Applied Biosystems) and the CFX96 Real-Time System (Bio-Rad) with gene-specific primers. Primers used for qPCR are listed and described in Table S1. The relative fold change in transcription was determined using the comparative C_T_ method and three housekeeping genes (*Gapdh*, *Rn18s* and *Actb*) as internal controls.

### ELISA

Maxisorb Immunolon 4HBX 96-well plates (Thermo Fisher Scientific) were used with mouse MCP-1 and mouse IL-1β ELISA kits (#432705 and #432605, respectively; BioLegend) according to the manufacturer's instructions to detect MCP-1 or IL-1β levels in plasma samples or medium from cell culture experiments. Colorimetric values were detected using a FLUOstar Omega plate reader.

### Statistical analysis

Data shown are mean±s.d. of *n* independent experiments or *n* animals (when necessary, *n* is reported in the corresponding figure legends). Statistical analyses were performed using GraphPad Prism 8. Data normality was determined by using the D'Agostino-Pearson omnibus test. Outliers were identified using the ROUT method (Q=1%). Statistical analyses, post-hoc tests and *P*-values are all described in corresponding figure legends. Significance was determined by a *P*-value of 0.05 or less. There were no statistically significant differences between male and female mice.

## Supplementary Material

Supplementary information
