# Supplementary material for: Cell-specific and athero-protective roles for RIPK3 in a murine model of atherosclerosis
Source: Dis Model Mech. 2020 Jan 24;13(1):dmm041962. doi: 10.1242/dmm.041962 (PMC6994951; doi:10.1242/dmm.041962)
Supplement: Supplementary information [file dmm-13-041962-s1.pdf]

# SUPPLEMENTAL MATERIAL

**Table S1. qPCR primers.** Primers used for analysis of transcripts in murine samples are listed.

| Gene | Forward (5' to 3') | Reverse (5' to 3') |
|---|---|---|
| *β-actin (Actb)* | TGTTACCAACTGGGACGACA | GGGGTGTTGAAGGTCTCAAA |
| *Casp1* | AGATGCCCACTGCTGATAGG | TTGGCACGATTCTCAGCATA |
| *Casp8* | GGGATACTGTCTGATCATCAAC | GGATAGGATACAGCAGATGAA |
| *Ccl2* | CCCACTCACCTGCTGCTACT | TCTGGACCCATTCCTTCTTG |
| *Cd68* | ACTTCGGGCCATGTTTCTCT | GCTGGTAGGTTGATTGTCGT |
| *Gapdh* | TCAACGGCACAGTCAAGG | ACTCCACGACATACTCAGC |
| *Icam1* | CAATTTCTCATGCCGCACAG | AGCTGGAAGATCGAAAGTCCG |
| *Il10* | ATTTGAATTCCCTGGGTGAGAAG | CACAGGGGAGAAATCGATGACA |
| *Il1b* | ACCAACAAGTGATATTCTCCATG | ATCCACACTCTCCAGCTGC |
| *Itgal* | AGATCGAGTCCGGACCCACAG | GGCAGTGATAGAGGCCTCCCG |
| *Itgb1* | GCAGGGCCAAATTGTGGGTGGT | GGCCGGAGCTTCTCTGCCAT |
| *Lyve1* | CTGGCTGTTTGCTACGTGAA | CGTCATCAGCCTTCTCTTCC |
| *Mlkl* | TCACAGATCTCCAGTTACCATC | ACGCAAGATGTTGGGAGAATCG |
| *Ripk1* | TACCTCCGAGCAGGTCAAAT | AAACCAGGACTCCTCCACAG |
| *Ripk3** | ACCCTTCAGAGGCACAACAC | TCCAGGGATACCAAGGAGTG |
| *Ripk3-Exon 10** | GCCAAGTATGACCAAGCACA | TTCAGGAAGTGGCAAGGACT |
| *Rn18s* | CCCGAAGCGTTTACTTTGAAA | CGCGGTCCTATTCCATTATTC |
| *Sele* | TGAACTGAAGGGATCAAGAAGACT | GCCGAGGGACATCATCACAT |
| *Sell* | CTCGAGGAACATCCTGAAGC | AATAAGGGCTTTTGGGCAAT |
| *Selp* | TCCAGGAAGCTCTGACGTACTTG | GCAGCGTTAGTGAAGACTCCGTAT |
| *Selplg* | AAGTGTCTGGCAGTGTGGAC | ATGGTACCGTGCCCAACAG |
| *Tlr4* | CCTGATGACATTCCTTCTTCAAC | TTGTTTCAATTTCACACCTGGATAAA |
| *Tnfa (Tnf)* | GTGATCGGTCCCCAAAGG | AGCCACTCCAGCTGCTCC |
| *Tnfr1 (Tnfrsf1a)* | GACCGGGAGAAGAGGGATAG | GTTCCTTTGTGGCACTTGGT |
| *Vcam1* | TGAACCCAAACAGAGGCAGAGT | GGTATCCCATCACTTGAGCAGG |

* Mouse *Ripk3-Exon 10* was detected using primers that hybridize to exon 10 of *Ripk3* only. By contrast, the full *Ripk3* transcript was detected using primers hybridizing to exons 8 and 9.

Disease Models & Mechanisms • Supplementary information

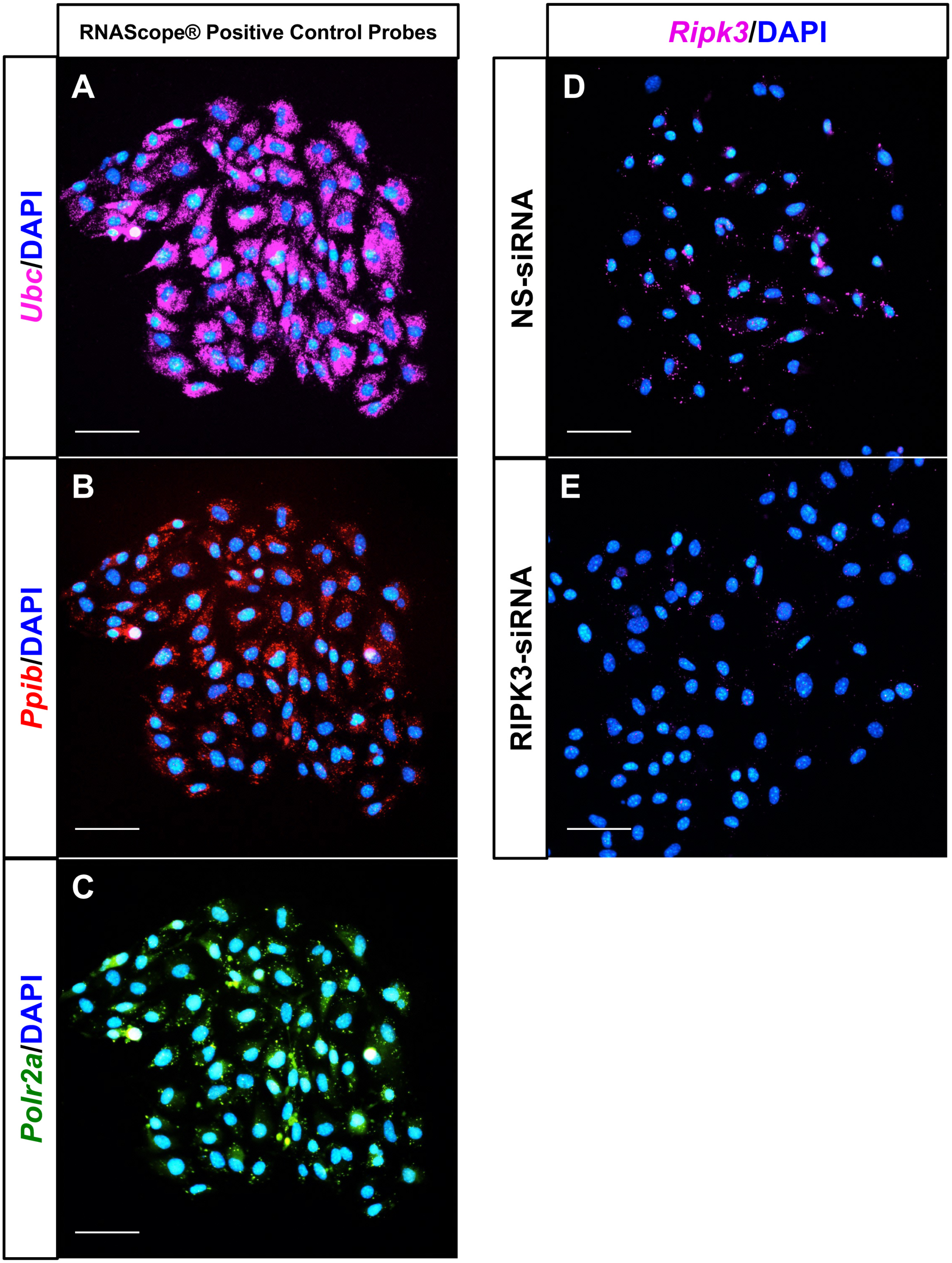
RNAScope® Positive Control Probes
Ripk3/DAPI
A
B
C
D
E
Ubc/DAPI
Ppib/DAPI
Polr2a/DAPI
NS-siRNA
RIPK3-siRNA

**Figure S1. Validation of the RNAScope® *Ripk3* probe.** (A-E): RNAScope® RNA in situ hybridization was performed on MS1 endothelial cells plated on chamber slides to test the specificity of the *Ripk3* probe. (A-C) Positive control probes for *Ubc* (pink; high transcript copy number), *Ppib* (red; medium transcript copy number), and *Polr2a* (green; low transcript copy number) were detected and costained for nuclei (DAPI; blue). (D,E) NS- or RIPK3-siRNA oligos were used to knockdown RIPK3 in MS1 cells while *Ripk3* probe was used to identify *Ripk3* transcripts (pink), and DAPI was used to identify nuclei (blue). Positive signal is determined by the presence of "dots", which are each meant to represent a single transcript. Scale bars: 25 µm.

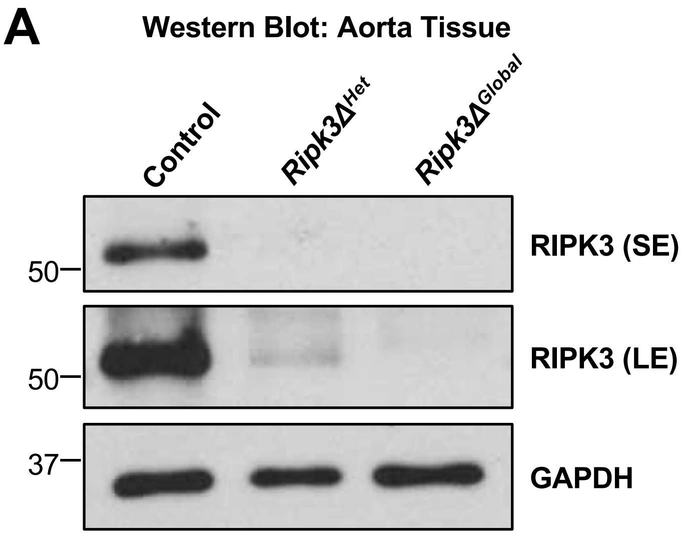


**Figure S2. RIPK3 protein levels in *Ripk3*-deficient aortas.** (A): Protein was collected from control (n=1), *Ripk3Δ*$^{Het}$ (n=1), and *Ripk3Δ*$^{Global}$ (n=1) aortas at 8 weeks of age. Protein lysates were immunoblotted to identify RIPK3 and GAPDH (loading control). SE: short exposure. LE: long exposure.

**A**

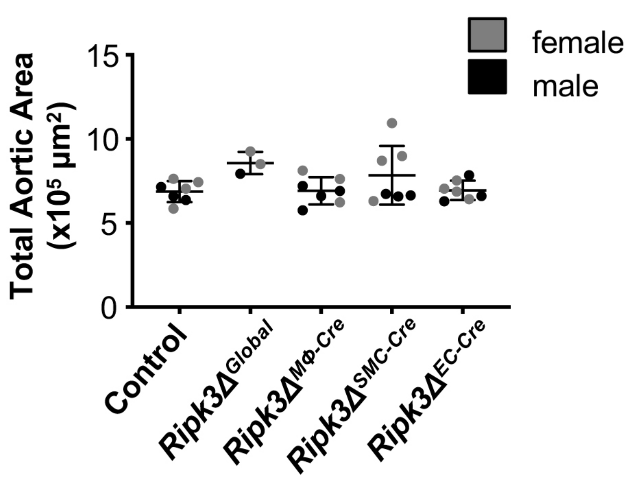


**B**

| Genotype | # of Aortic Roots with Calcification | Total # of Aortic Roots Analyzed |
|---|---|---|
| Control | 1 (F) | 7 |
| *Ripk3Δ*$^{Global}$ | 0 | 3 |
| *Ripk3Δ*$^{MΦ\text{-}Cre}$ | 1 (F) | 7 |
| *Ripk3Δ*$^{SMC\text{-}Cre}$ | 0 | 7 |
| *Ripk3Δ*$^{EC\text{-}Cre}$ | 1 (F) | 7 |

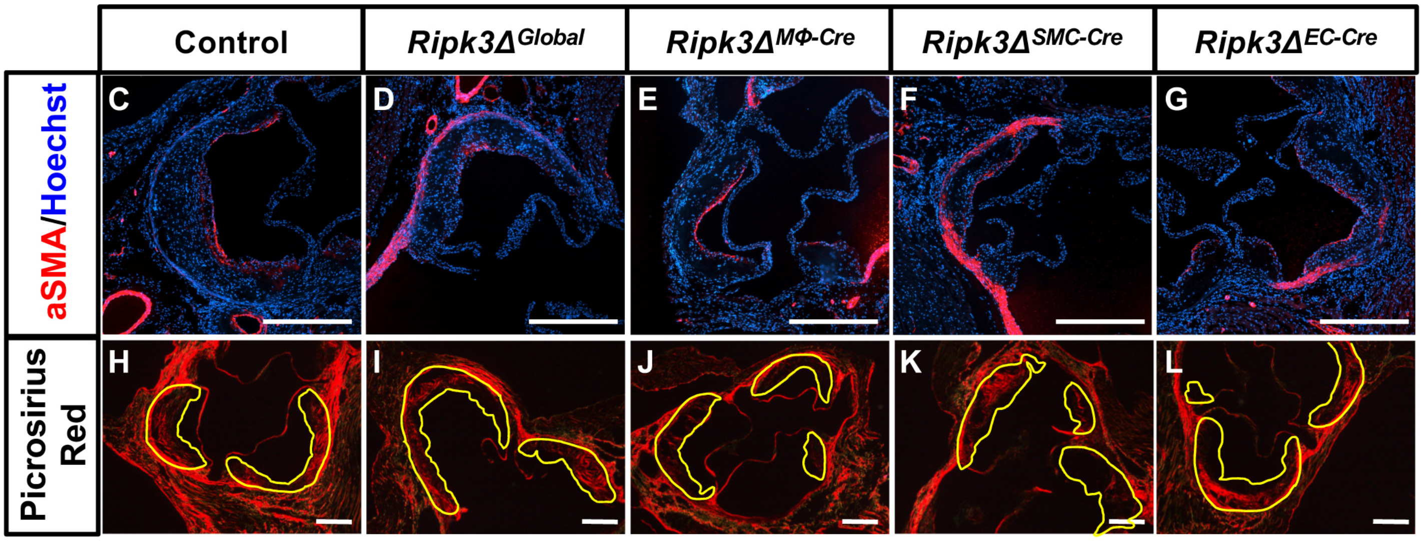


**M**

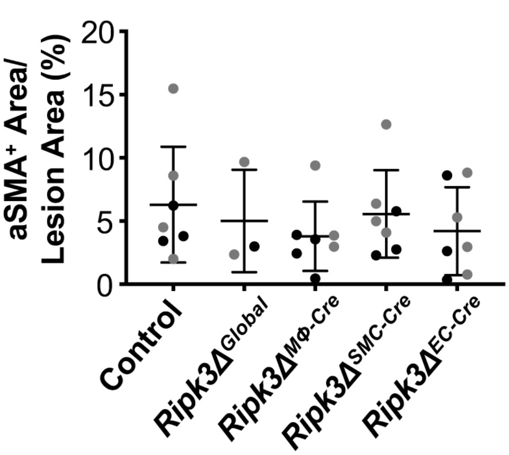


**N**

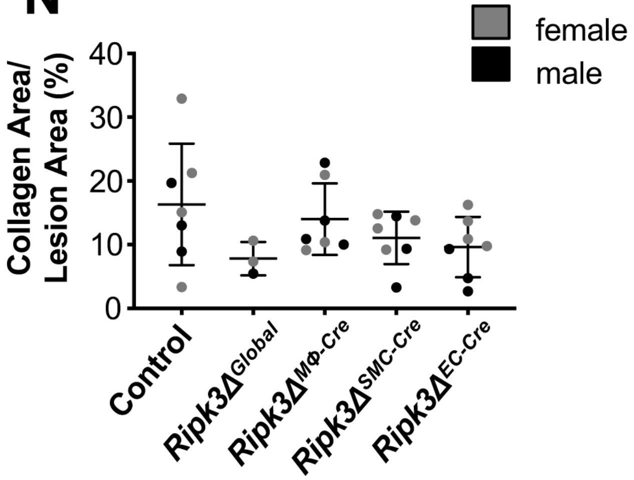


Disease Models & Mechanisms • Supplementary information

**Figure S3. Aortic root analyses reveal no differences in total aortic area, smooth muscle cell area, or collagen area.** (A): After 3 months on a Western diet, hearts were dissected, and aortic roots were sectioned and stained with Oil Red O and hematoxylin to visualize lesion area. Total aortic area was measured in order to normalize the lesion area of each sample and to confirm that the total aortic area did not vary. (B): Aortic roots were stained with Alizarin Red S to visualize calcification. Calcification was reported as either being present or absent and is displayed in a table format (F indicates female). (C-N): Aortic roots were analyzed for plaque stability by using markers for smooth muscle cells or collagen. (C-G,M) Aortic roots were immunostained for aSMA (red) and nuclei (Hoechst; blue) to visualize smooth muscle cell infiltration into the plaque. Smooth muscle cell area was defined as the aSMA$^+$ area of the intima. The media was excluded. Smooth muscle cell area was reported as the aSMA$^+$ percent of the lesion area. (H-L,N) Aortic roots were stained with Picrosirius Red and visualized under polarized light. Collagen area was defined as the red, green, and yellow signal seen in the plaque regions (outlined in yellow). Collagen area was reported as the percent collagen of the lesion area. For panels A, M, and N, each dot represents an individual animal. Statistics were calculated using one-way ANOVA. Overall p-values are (A) 0.08, (M) 0.71, and (N) 0.18. Bar graphs represent mean $\pm$ SD. Scale bars: 200 μm.

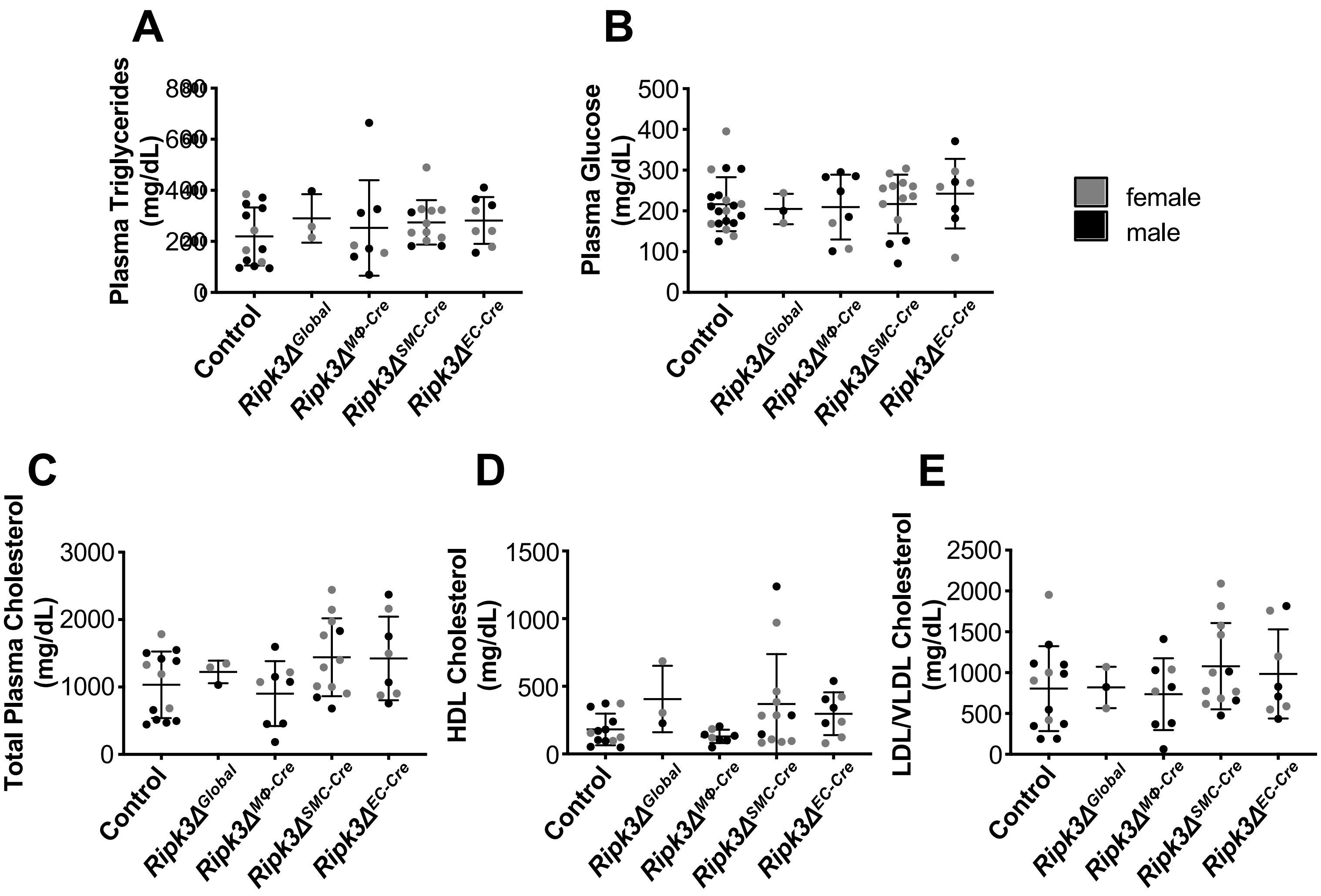


**Figure S4. Plasma triglyceride, glucose, and cholesterol levels are unchanged between the genotypes.** (A-E): After mice were fed a Western diet for 3 months, plasma was collected and analyzed for (A) triglycerides, (B) glucose, (C) total cholesterol, (D) HDL cholesterol, and (E) LDL/VLDL cholesterol. Each dot represents an individual animal. Statistics were calculated using Kruskal-Wallis tests for A-D and one-way ANOVA for E. Overall p-values are (A) 0.44, (B) 0.80, (C) 0.30, (D) 0.09, and (E) 0.54. Bar graphs represent mean±SD.

Disease Models & Mechanisms • Supplementary information

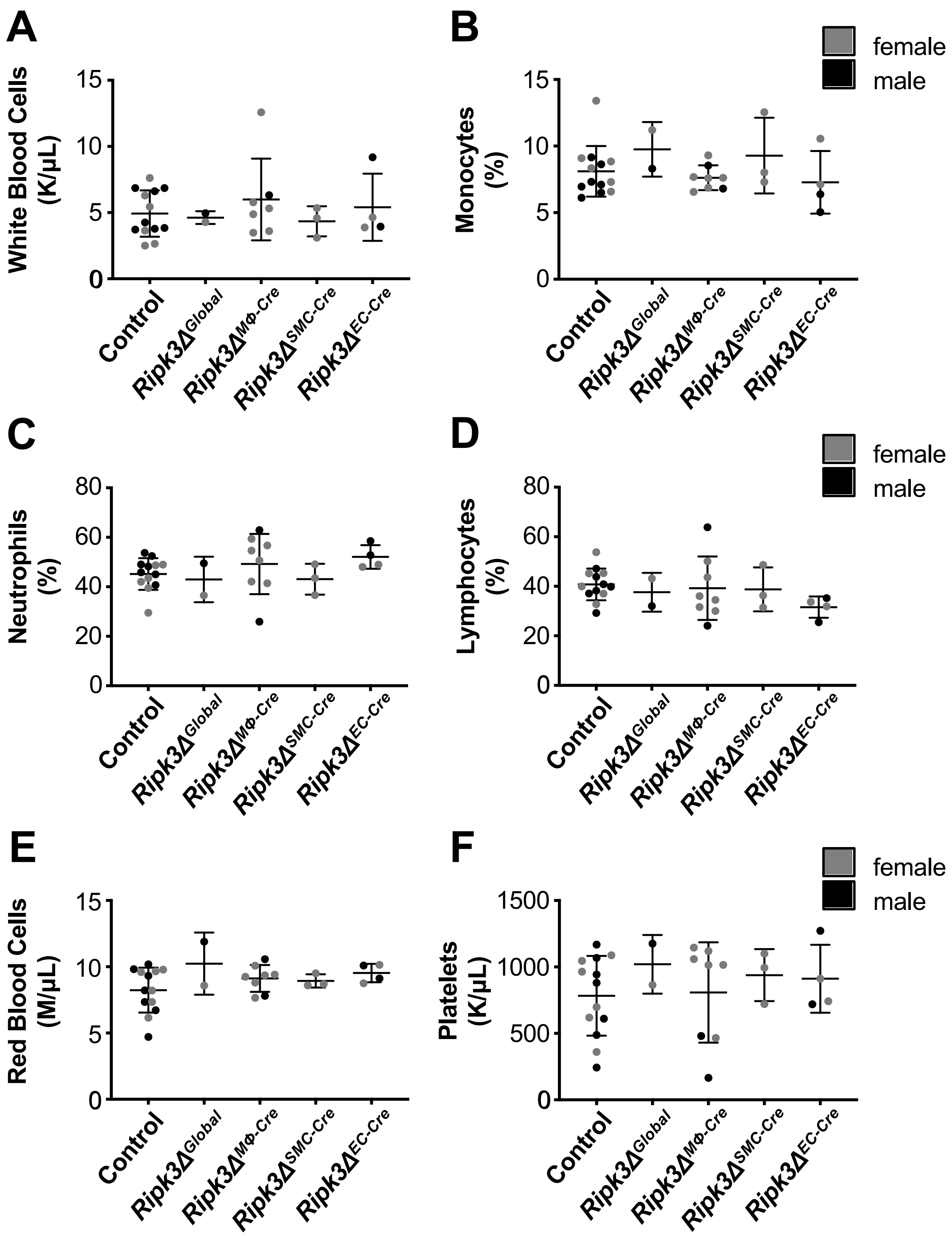


**Figure S5. Blood cell counts are unchanged between the genotypes.**(A-F): After mice were fed a Western diet for 3 months, blood was collected and analyzed for (A) white blood cells, (B) percent monocytes, (C) percent neutrophils, (D) percent lymphocytes, (E) red blood cells, and (F) platelets. Each dot represents an individual animal. Statistics were calculated using a Kruskal-Wallis test for B and one-way ANOVA for A,C,D,E,F. Overall p-values are (A) 0.78, (B) 0.43, (C) 0.46, (D) 0.50, (E) 0.25, and (F) 0.79. Bar graphs represent mean±SD.

Disease Models & Mechanisms • Supplementary information

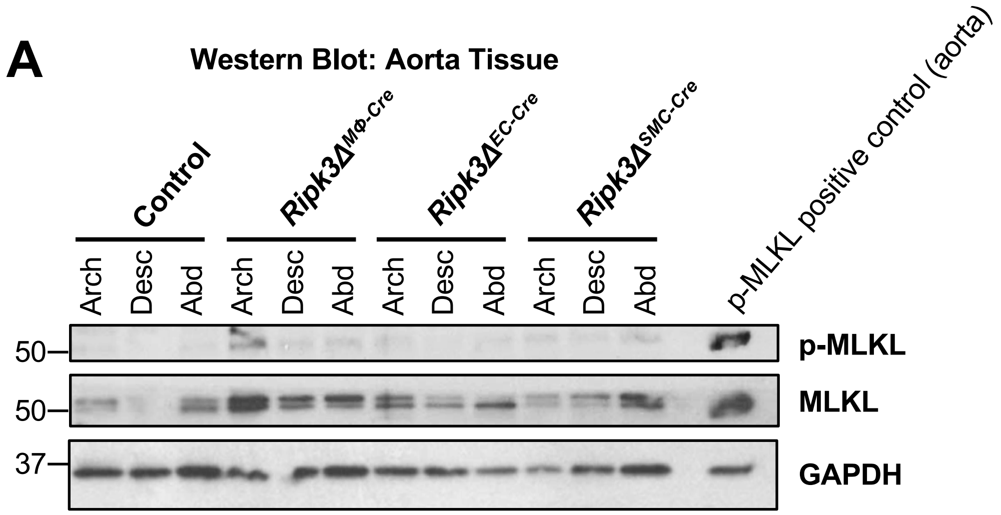


**Figure S6. p-MLKL levels are nearly undetectable in advanced plaques (alternative antibody).** (A): After 3 months on a Western diet, protein was collected from control (n=1), *Ripk3Δ*$^{MΦ\text{-}Cre}$ (n=1), *Ripk3Δ*$^{EC\text{-}Cre}$ (n=1), and *Ripk3Δ*$^{SMC\text{-}Cre}$ (n=1) aortas. Protein lysates were immunoblotted to identify p-MLKL (Cell Signaling Technology; #37333), MLKL, and GAPDH (loading control). The p-MLKL positive control aorta was collected from a non-atherosclerotic, yet severely ill animal.

Disease Models & Mechanisms • Supplementary information

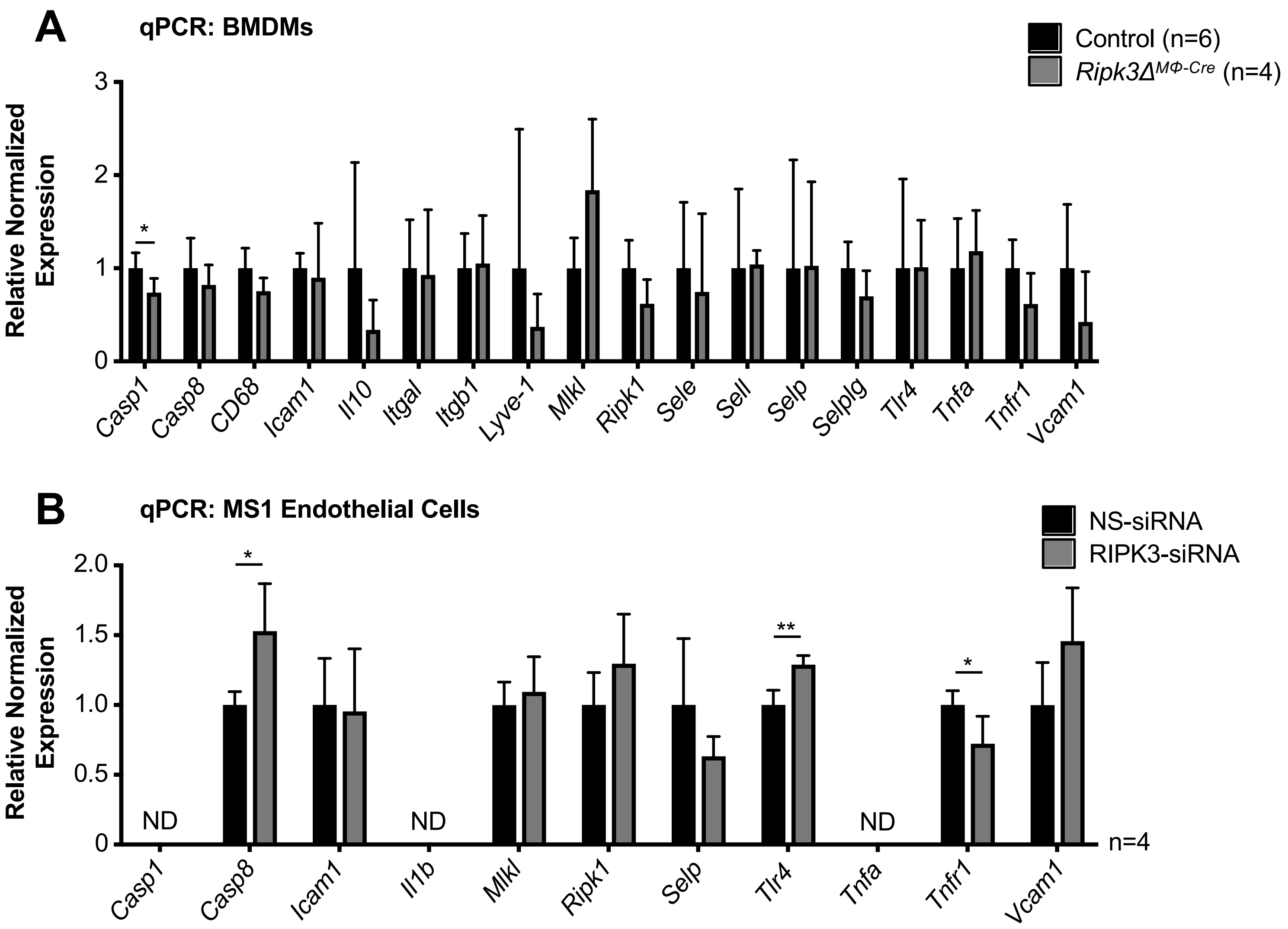


**Figure S7. Genes from *Ripk3*-deficient BMDMs and MS1 endothelial cells that are not influenced by RIPK3.** (A): BMDMs were isolated from control (n=6) and *Ripk3Δ*$^{MΦ\text{-}Cre}$ (n=4) bone marrow, and RNA was collected 7 days later. Each *n* value is a BMDM isolation from an individual animal. RNA was converted to cDNA and analyzed by qPCR using primers described in Table S1. (B): MS1 endothelial cells were transfected with NS- and RIPK3-siRNA oligos for 24 hrs and were then cultured in low-serum medium for another 24 hrs before RNA was collected, converted to cDNA, and analyzed by qPCR (n=4 independent experiments). Significant changes in expression are indicated here; however, the changes were minor and thus we deemed them as unworthy of further exploration. Statistics were calculated using unpaired t-tests, with Welch's correction when necessary. (*) indicates p<0.05. (**) indicates p<0.01. ND indicates Not Detected. Bar graphs represent mean±SD.

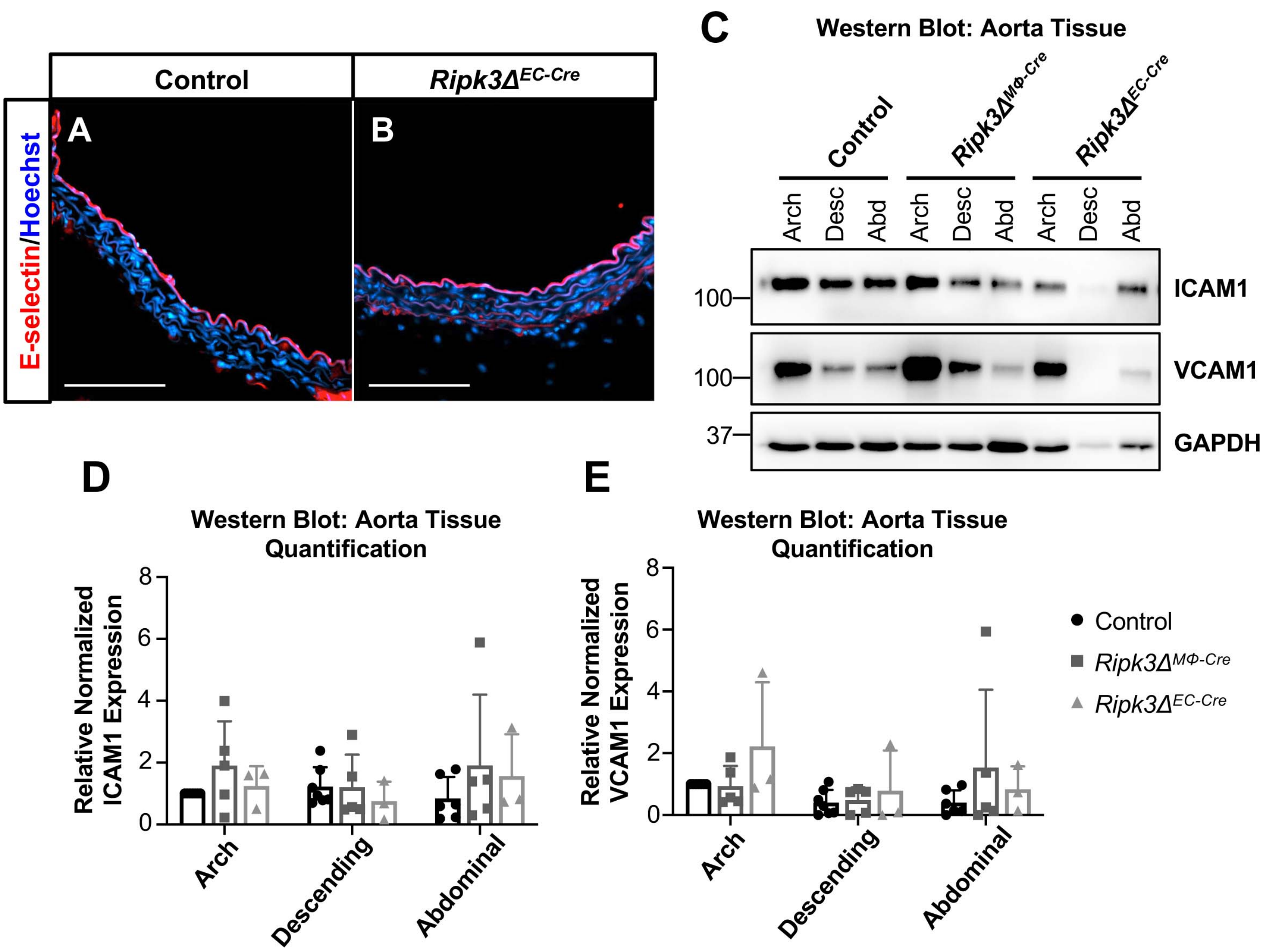


**Figure S8. E-selectin immunostaining intensity does not differ between control and *Ripk3Δ*$^{EC\text{-}Cre}$ aortas, while ICAM1 and VCAM1 levels are unaffected by *Ripk3* deletion.** (A,B): After 3 months on a Western diet, the descending aortas of control (n=3) and *Ripk3Δ*$^{EC\text{-}Cre}$ (n=1) mice were embedded, sectioned, and immunostained for E-selectin (red) and nuclei (Hoechst, blue). (C-E): Protein was collected from control (n=7), *Ripk3Δ*$^{MΦ\text{-}Cre}$ (n=5), and *Ripk3Δ*$^{EC\text{-}Cre}$ (n=3) aortas. Protein lysates were immunoblotted to identify ICAM1, VCAM1, and GAPDH (loading control) and quantified. For panels D and E, each dot represents an individual animal. Statistics were calculated using two-way ANOVA. Overall p-values are (D) 0.24 and (E) 0.29. Bar graphs represent mean±SD. Scale bars: 50 μm.

Disease Models & Mechanisms • Supplementary information

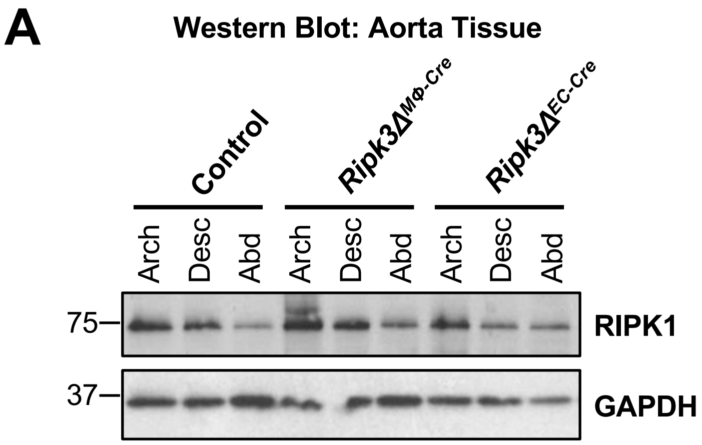


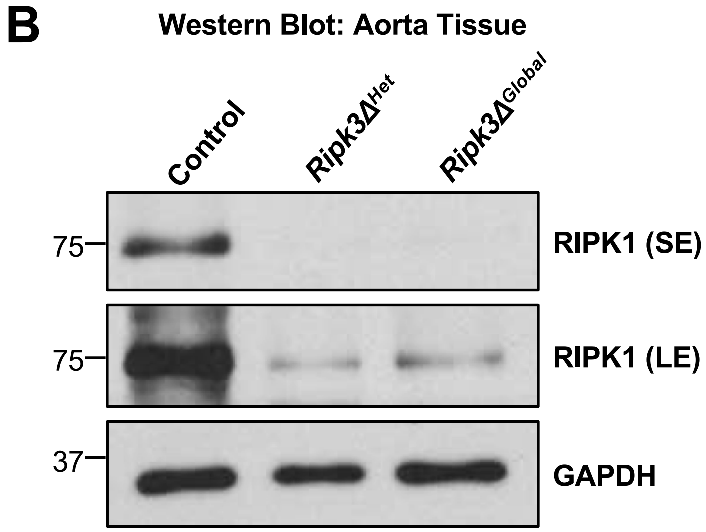


**Figure S9. RIPK1 levels are decreased in heterozygous and global *Ripk3*-deficient aortas.** (A): After 3 months on a Western diet, protein was collected from control (n=1), *Ripk3Δ*$^{MΦ\text{-}Cre}$ (n=1), and *Ripk3Δ*$^{EC\text{-}Cre}$ (n=1) aortas. Protein lysates were immunoblotted to identify RIPK1 and GAPDH (loading control). RIPK1 levels were not noticeably affected by cell-specific *Ripk3* deletion. Note that the same transfer membrane used for detecting p-MLKL and MLKL (in Fig. S6A) was reused for this RIPK1 blot; the GAPDH blots are the same in both figures. (B): Protein was collected from control (n=1), *Ripk3Δ*$^{Het}$ (n=1), and *Ripk3Δ*$^{Global}$ (n=1) aortas at 8 weeks of age. Protein lysates were immunoblotted to identify RIPK1 and GAPDH (loading control). RIPK1 is substantially decreased when either one or both alleles of RIPK3 are deleted globally. SE: short exposure. LE: long exposure.

Disease Models & Mechanisms • Supplementary information

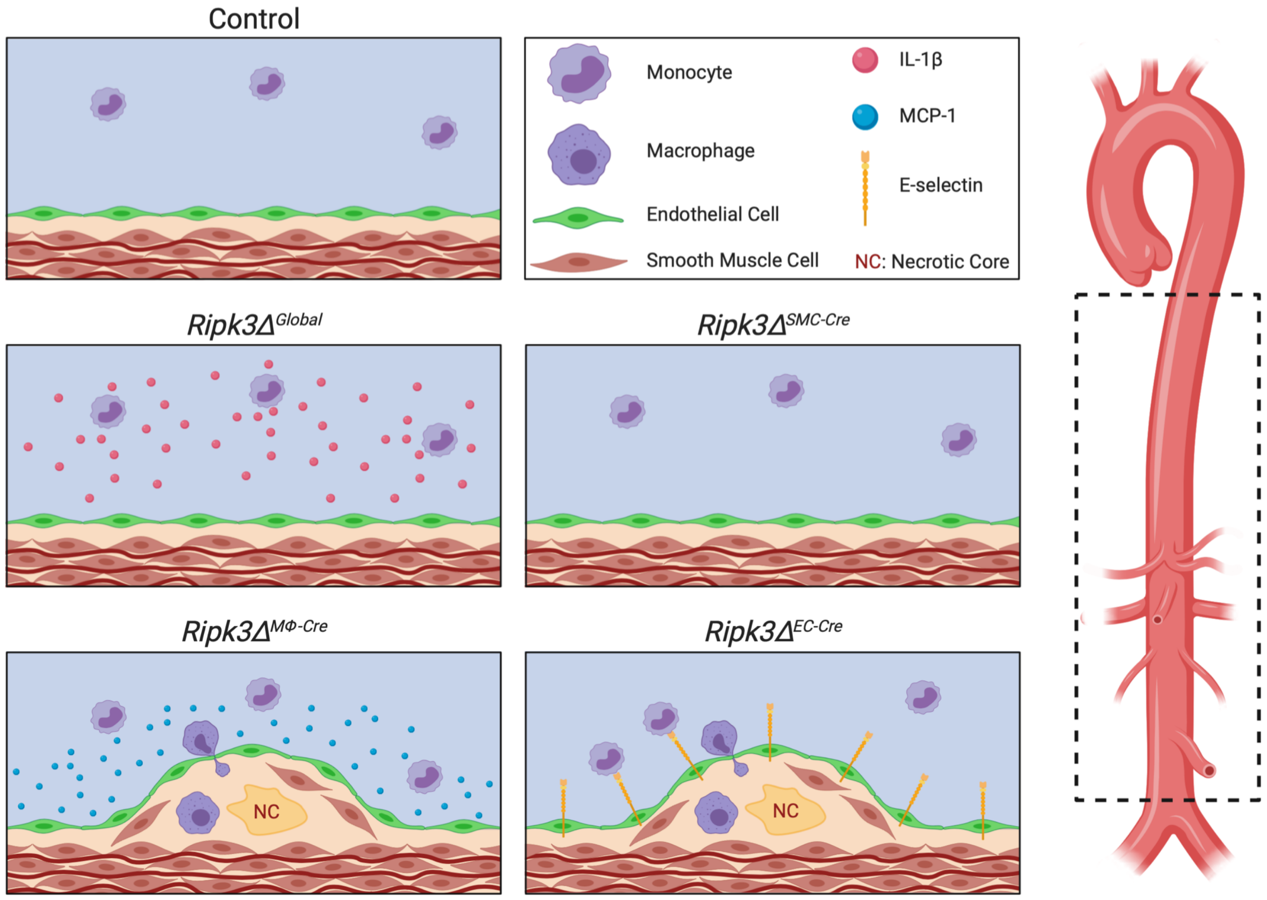


**Figure S10. Data Summary Model.** This model summarizes data related to atherosclerotic plaque burden in the descending and abdominal regions of mouse aortas (boxed region of aorta cartoon at right) with global and cell-specific deletion of *Ripk3*. After 3 months on a Western diet, plaque burden was low in the descending and abdominal regions of control aortas. Lesion area was unaffected by *Ripk3*-deficiency at a global level or in smooth muscle cells. However, IL-1β levels were significantly increased in the global knockout. The descending and abdominal aortic regions contained more lesion areas in mice with *Ripk3*-deficiency in macrophages or endothelial cells, which could be due to an increase in MCP-1 and E-selectin, respectively. Abdominal aortas with *Ripk3*-deficient macrophages expressed more MCP-1, a chemokine that attracts circulating leukocytes. Likewise, aortas with *Ripk3*-deficient endothelial cells show an upward trend in E-selectin, which is a surface receptor that assists in leukocyte recruitment. Created with BioRender.com.

Disease Models & Mechanisms • Supplementary information
